# Novel Mutations Detected in Avirulence Genes Overcoming Tomato *Cf* Resistance Genes in Isolates of a Japanese Population of *Cladosporium fulvum*

**DOI:** 10.1371/journal.pone.0123271

**Published:** 2015-04-22

**Authors:** Yuichiro Iida, Pieter van ‘t Hof, Henriek Beenen, Carl Mesarich, Masaharu Kubota, Ioannis Stergiopoulos, Rahim Mehrabi, Ayumi Notsu, Kazuki Fujiwara, Ali Bahkali, Kamel Abd-Elsalam, Jérôme Collemare, Pierre J. G. M. de Wit

**Affiliations:** 1 National Agriculture and Food Research Organization, Tsu, Mie, Japan; 2 Wageningen University, Laboratory of Phytopathology, Wageningen, The Netherlands; 3 Seed and Plant Improvement Institute, Karaj, Iran; 4 Hokkaido Research Organization, Ornamental Plants and Vegetables Research Center, Hokkaido, Japan; 5 King Saud University, College of Science, Botany and Microbiology Department, Riyadh, Saudi Arabia; 6 Plant Pathology Research Institute, Agricultural Research Center, Giza, Egypt; 7 Centre for Biosystems Genomics, Wageningen, The Netherlands; Agriculture and Agri-Food Canada, CANADA

## Abstract

Leaf mold of tomato is caused by the biotrophic fungus *Cladosporium fulvum* which complies with the gene-for-gene system. The disease was first reported in Japan in the 1920s and has since been frequently observed. Initially only race 0 isolates were reported, but since the consecutive introduction of resistance genes *Cf-2*, *Cf-4*, *Cf-5* and *Cf-9* new races have evolved. Here we first determined the virulence spectrum of 133 *C*. *fulvum* isolates collected from 22 prefectures in Japan, and subsequently sequenced the avirulence (*Avr*) genes *Avr2*, *Avr4*, *Avr4E*, *Avr5* and *Avr9* to determine the molecular basis of overcoming *Cf *genes. Twelve races of *C*. *fulvum* with a different virulence spectrum were identified, of which races 9, 2.9, 4.9, 4.5.9 and 4.9.11 occur only in Japan. The *Avr* genes in many of these races contain unique mutations not observed in races identified elsewhere in the world including (i) frameshift mutations and (ii) transposon insertions in *Avr2*, (iii) point mutations in *Avr4 *and *Avr4E*, and (iv) deletions of *Avr4E*, *Avr5* and *Avr9*. New races have developed by selection pressure imposed by consecutive introductions of *Cf-2*, *Cf-4*, *Cf-5 *and *Cf-9* genes in commercially grown tomato cultivars. Our study shows that molecular variations to adapt to different *Cf* genes in an isolated *C*. *fulvum* population in Japan are novel but overall follow similar patterns as those observed in populations from other parts of the world. Implications for breeding of more durable *C*. *fulvum* resistant varieties are discussed.

## Introduction

*Cladosporium fulvum* Cooke [syn. *Passalora fulva* (Cooke) U. Braun & Crous] is a biotrophic pathogen that causes leaf mold of tomato [1]. The fungus has been reported on tomato since the late 1800s [2]. The disease is primarily a problem in greenhouse-grown tomatoes and occurs worldwide in areas with high humidity and moderate temperatures. Infection begins with conidia germinating on the lower leaf surface and producing runner hyphae that enter the host through stomata. Subsequently, the fungus colonizes the intercellular space between mesophyll cells, and 10–14 days after penetration, conidiophores emerge from stomata producing large number of conidia that can re-infect tomato leaves [1, 3–5].

The *C*. *fulvum*-tomato interaction follows the gene-for-gene system indicating that each dominant pathogen avirulence (*Avr*) gene product is recognized by the product of a corresponding dominant host *Cf* resistance gene directly or indirectly [6]. To date, five *Avr* genes (*Avr2*, *Avr4*, *Avr4E*, *Avr5*, and *Avr9*) have been cloned and characterized from *C*. *fulvum* [7–13], and their encoded proteins trigger a hypersensitive response (HR) in host plants carrying the corresponding *Cf-2*, *Cf-4*, *Cf-4E*, *Cf-5*, and *Cf-9* genes, respectively [14–18]. The different *Cf*-genes encoding leucine-rich receptor-like proteins, originate from wild *Solanum* species, and have been introduced into tomato cultivars currently grown worldwide [19]. However, by selection pressure imposed by *Cf* genes, new *C*. *fulvum* races evolved that overcome introduced *Cf* resistance genes. DNA modifications observed in *Avr* genes of new races resulted in frame-shift mutations or point mutations leading to amino acid substitutions in the encoded Avr proteins, whereas complete loss of an *Avr* gene and transposon insertions in *Avr* genes were also observed [11, 20–23].

In Japan, the first reports on the occurrence of tomato leaf mold date from the 1920s [24]. Initially, race 0 isolates carrying all known *Avr* genes were the only indigenous isolates identified and there were no reports of pathogen specialization until the 1960s [24]. In the USA, Canada and Western Europe, breeding of tomato cultivars resistant against leaf mold started in the 1930s [25, 26] and in Japan in the 1960s. In Japan, the *Cf-2* gene was the first resistance gene to be introduced in commercial tomato lines in 1965 [27]. As a consequence race 2 isolates were identified in the late 1970s [28]. Soon after the introduction of the *Cf-4* gene (and likely also the *Cf-11* gene) in the 1990s, races 2.4 and 2.4.11 isolates appeared [29] followed by race 4 and 4.11 isolates in 2003 [30]. In the 2000s, new resistant cultivars carrying the *Cf-9* gene were launched, and in 2008, the races 4.9, 4.9.11 and 2.9 isolates that overcome *Cf-9*-mediated resistance were identified [31, 32]. It is not known when the *Cf-5* gene was introduced, but recently, in Japan, new races (2.5.9 and 4.5.9) were identified on a cultivar carrying the *Cf-5* and *Cf-9* genes, though the presence of these genes in this cultivar was then unknown [33]. Of the eleven races presently identified in Japan (races 0, 2, 2.4, 2.4.11, 2.9, 2.5.9, 4, 4.9, 4.5.9, 4.11, 4.9.11), seven also occur elsewhere in the world [34], whereas races 2.9, 4.9, 4.5.9 and 4.9.11 are unique to Japan [31–33].

For many years *C*. *fulvum* was not a serious economic problem for tomato growers in Japan, but the recent appearance of new races [31–33] prompted us to perform a detailed study of the virulence spectrum of the fungal population in the whole country and to analyze the molecular basis of adaptation to the introduced *Cf* genes. Of all eleven races of *C*. *fulvum* in Japan nothing is known about DNA modifications in *Avr* genes that cause adaptation to the corresponding *Cf* resistance genes. To understand the molecular basis of adaptation of *C*. *fulvum* to the introduced *Cf* genes in Japan, we determined both the virulence spectrum and the DNA modifications present in the *Avr* genes of 133 isolates of a *C*. *fulvum* population collected between 1997 and 2013. Most of the new races appeared to be confined to particular regions of the country, whereas older ones have spread over the whole country. We identified many new DNA modifications in *Avr* genes leading to virulence on plants with corresponding *Cf* genes that are unique to the Japanese *C*. *fulvum* population. We determined the effect of new DNA modifications on HR-inducing activities of *Avr2*, *Avr4* and *Avr5* genes present in Japanese races of *C*. *fulvum* on tomato plants carrying the corresponding *Cf* resistance gene. For some of the new races, the parental isolate(s) of new races could be inferred based on shared sequences in *Avr* genes.

## Materials and Methods

### Ethics Statement

**Fig 1:**
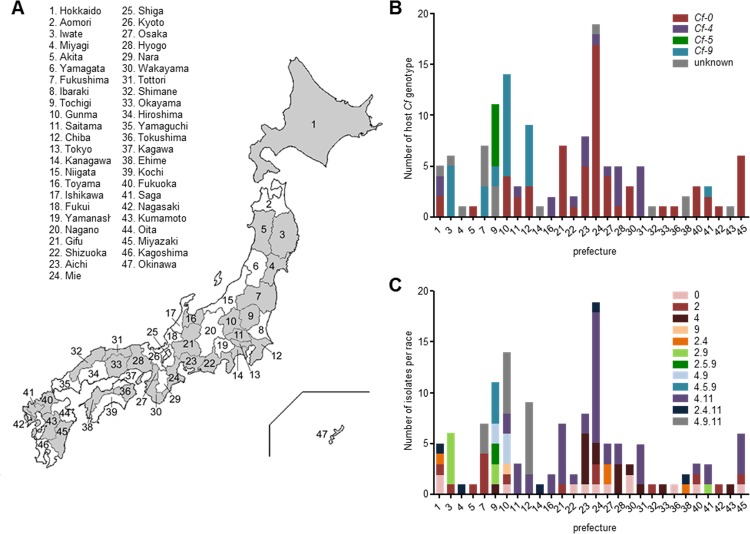
Distribution of tomato *Cf* resistance genes and *Cladosporium fulvum* races in Japan. Prefectures from where *Cladosporium fulvum* isolates were collected (**A**), the distribution of *Cf* genes employed in the different prefectures (**B**), and the virulence spectrum of the isolates collected in these prefectures (**C**) are presented. The surveys were conducted between 1997 and 2013 in prefectures highlighted in grey. The prefecture numbers correspond to those shown in S2 Table.

Between 1997 and 2013, diseased tomato leaves, from which the *C*. *fulvum* were isolated, were collected from 22 of Japan’s 47 prefectures (Fig 1A). Sampling of tomato leaves was performed in private greenhouses under the permission by all owners. No specific permissions were required for the all locations. The surveys did not involve regulated, endangered, or protected species.

### Fungal isolates and virulence assays

Collected leaves showed the typical leaf mold symptoms: pale-green or yellow spots on the upper side, and multiple velvet, olive-brown conidia on the lower side of the leaves. 133 single-spores isolated from these lesions were cultured on potato dextrose agar (PDA) for two weeks at 20ºC. Isolates were collected from greenhouses at different locations in these prefectures. There is a slight over-representation of isolates collected from Gunma and Mie prefectures, where recent outbreaks of leaf mold were reported. When available, information on the *Cf* genes present in the tomato cultivars from which the isolates were collected was recorded. The virulence spectrum of the isolates was determined by inoculating them on a differential set of tomato cultivars including ‘Potentate’ (no *Cf* resistance gene), ‘Vetomold’ (*Cf-2*), ‘Purdue 135’ (*Cf-4*), ‘Moneymaker-Cf-5’ (*Cf-5*), ‘Ontario 7818’ (*Cf-6*), ‘Moneymaker-Cf-9’ (*Cf-9*), and ‘Ontario 7716’ (*Cf-4* and *Cf-11*). Three four-week-old plants of each differential cultivar were spray-inoculated on the lower side of the leaves with a conidial suspension of 10^4^ spores/ml of each isolate. The inoculated plants were incubated in a moist chamber at 100% humidity and 25°C with a 16 h light/8 h dark photoperiod. After two to three weeks, the inoculated plants were analyzed and scored visually as either resistant or susceptible. Susceptible cultivars showed heavy sporulation, whereas resistant cultivars were immune and free of disease symptoms. A representative set of 120 isolates was submitted to the microorganism component of the GeneBank resources maintained by the National Institute of Agrobiological Sciences (http://www.gene.affrc.go.jp/index_en.php) under accession numbers MAFF 242495 to 242550, MAFF 242556 to 242575, and MAFF731146 to 731149.

### DNA manipulation and sequencing

DNA was isolated from 133 isolates of *C*. *fulvum* for analysis of DNA modifications in *Avr* genes. In addition, the DNA sequence of *Avr* genes in four Japanese reference isolates CF5, CF9, CF44 and CF56 collected in Japan in 1973 was also determined [29, 35]. Mycelium from the isolates grown on PDA was collected, transferred to Eppendorf tubes, freeze-dried overnight and disrupted in liquid nitrogen using the Retsch Qiagen Tissue Lyser twice for 30 s with 30 oscillations per minute. DNA was extracted using the DNeasy Plant Mini Kit (Qiagen, USA) according to the manufacturer’s instructions. The DNA concentration was determined using a Nanodrop ND-1000 spectrophotometer (Thermo Scientific, USA).

The mating type in the collection of 133 isolates was examined by PCR amplification of partial genes (MAT1-1 or MAT1-2) using the primers shown in S1 Table. PCR reactions were performed in 25μl volumes containing 50 ng genomic DNA, 1× GoTaq PCR buffer, 0.2 mM dNTPs, 0.4 μM of each primer and 1 U of GoTaq DNA polymerase (Promega, USA). The PCR conditions were 95ºC for 2 min, followed by 35 cycles of 95 ºC for 30 s, 60 ºC for 30 s and 72 ºC for 1 min, and a final extension at 72 ºC for 5 min. The PCR products obtained were separated by electrophoresis and visualized under UV-light.

PCR reactions for amplification of the *Avr2*, *Avr4*, *Avr4E*, *Avr5* and *Avr9* genes were performed with the primers shown in S1 Table. PCR reactions were performed in 25μl volumes containing 50 ng genomic DNA, 1× GoTaq PCR buffer, 0.2 mM dNTPs, 0.4 μM of each primer and 1 U of GoTaq DNA polymerase (Promega, USA). The PCR conditions were 95ºC for 2 min, followed by 35 cycles of 95 ºC for 30 s, 60 ºC for 30 s and 72 ºC for 1 min, and a final extension at 72 ºC for 7 min. The PCR products were purified using Illustra GFX PCR DNA and Gel Band Purification Kit (GE Healthcare) according to the manufacturer´s instructions. After DNA purification the DNA concentration was measured using a Nanodrop spectrophotometer. Purified PCR products were sequenced by Macrogen Inc. (Seoul, South-Korea). DNA sequences of the *Avr* genes were analyzed using the Lasergene package (DNASTAR, USA) and compared with the sequences present in four Japanese reference isolates collected in 1973 as well as those present in a worldwide collection of *C*. *fulvum* isolates [21]. The DNA sequence of the *Avr* genes in the four reference isolates were considered as wild-type.

### PVX-mediated expression of *Avr* genes in tomato plants with corresponding *Cf* genes

In order to confirm that the DNA modifications observed in the collected isolates were the cause of overcoming a particular *Cf*-gene, we investigated the HR-inducing activities of wild-type and unique mutant versions of *Avr2*, *Avr4* and *Avr5* genes present in Japanese races of *C*. *fulvum* on tomato plants carrying the matching *Cf* resistance gene. To this end, we expressed them in tomato plants carrying corresponding *Cf* genes by agroinfection using pSfinx, a modified binary Potato Virus X (PVX)–based vector for transient expression of foreign genes into plants as described by Stergiopoulos *et al*. [36]. This system is based on *Agrobacterium tumefaciens*-mediated delivery of the recombinant PVX virus that enables targeted systemic production of Avr proteins into the apoplast of PVX-infected plants. The wild-type and mutant versions of *Avr2*, *Avr4* and *Avr5* genes were cloned into the PVX vector using primers presented in S1 Table. Ten-day-old tomato seedlings were agroinfected and analyzed by a method described by van der Hoorn *et al*.[37]. Photographs were taken at 20 days post inoculation. HR-inducing activities of *Avr4E* and *Avr9* were not assayed as the observed mutations leading to virulence observed in these genes involved loss of the complete gene or a point mutation that was already reported to overcome a particular *Cf* gene (*Cf-4E* and *Cf-9*, respectively). Thus, only *Avr* genes with unique new mutations were tested for loss of their HR-inducing activity.

## Results

### The virulence spectrum of isolates of a *Cladosporium fulvum* population collected in Japan

Morphological characteristics of single-spore isolates and disease symptoms caused by them after inoculation onto susceptible tomato plants appeared similar to those described previously for *C*. *fulvum* [38]. In total 133 *C*. *fulvum* single-spore isolates were collected from diseased tomato plants grown in Japan from north to south (Fig 1A). For each isolate, its acronym, year of sampling, prefecture, and the *Cf* resistance gene present in the cultivar from which the isolate was collected, were recorded (S2 Table). In addition, the mating type (MAT1-1 or MAT1-2) of all isolates was determined because both are still present in worldwide populations although *C*. *fulvum* is supposed to be an asexual fungus [21, 39]. Both mating types are also present in Japan, with a slight bias for MAT1-2 (73%; S2 Table).

**Fig 2:**
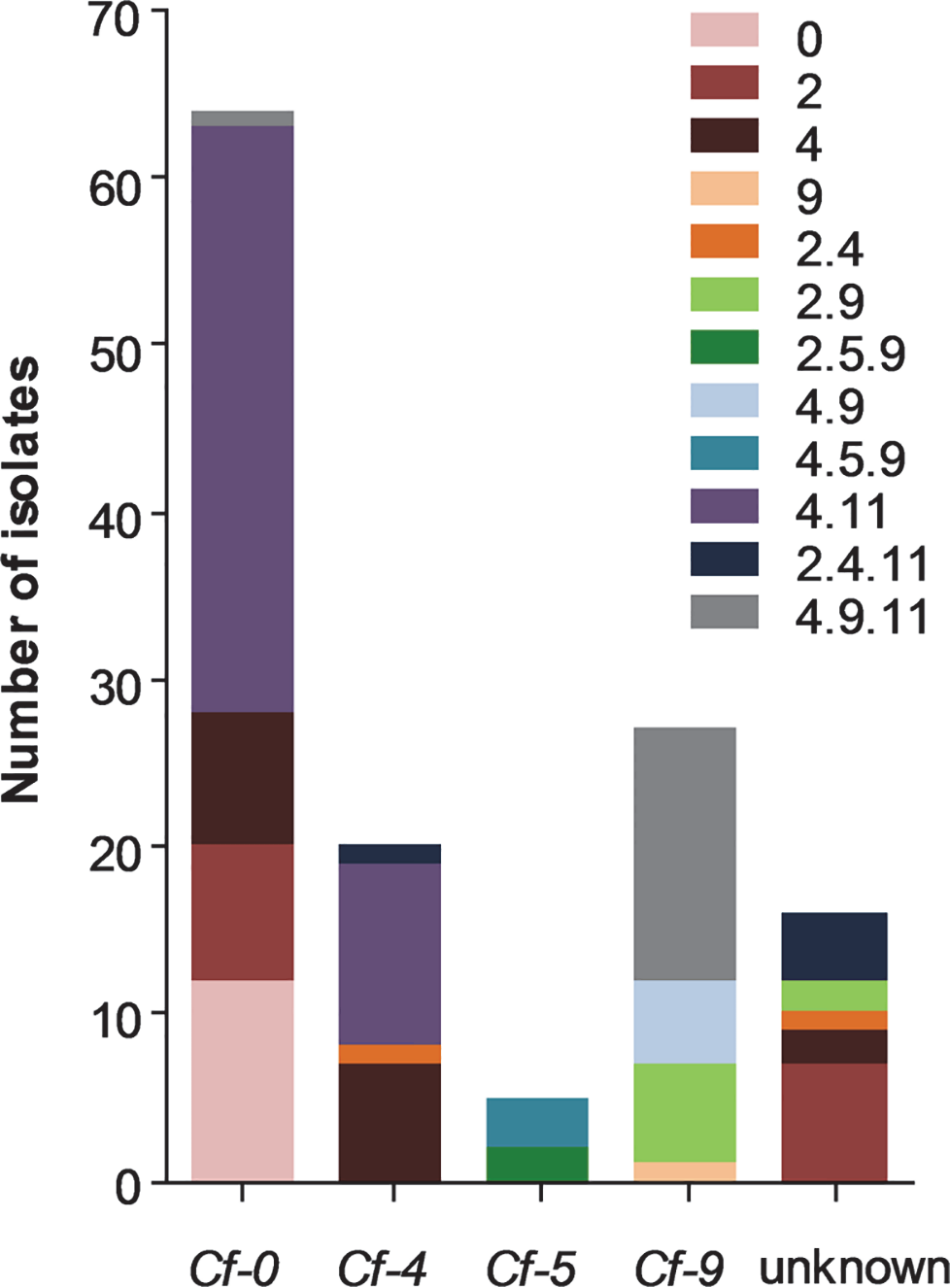
The frequency of *Cladosporium fulvum* races collected from tomato cultivars carrying no *Cf* resistance gene (*Cf-0*), the *Cf-4* or *Cf-9* resistance gene, or from tomato cultivars of which the *Cf* gene was not known.

The virulence spectrum of the 133 *C*. *fulvum* isolates is shown in Table 1. In total twelve races with a novel virulence spectrum were identified: races 0, 2, 4, 9, 2.4, 2.9, 2.5.9, 4.9, 4.5.9, 4.11, 2.4.11, and 4.9.11. It is the first time a race 9 is reported in Japan (prefecture Gunma) and elsewhere in the world. The frequencies of the different races in the different prefectures vary obviously, which most likely reflects the different frequencies of the *Cf-4*, *Cf-5* and *Cf-9* genes employed in these prefectures (Fig 1B and 1C). Indeed, all isolates that overcome *Cf-5* and *Cf-9* resistance were isolated from *Cf-5* and *Cf-9* plants, respectively, introduced in prefectures Iwate, Fukushima, Tochigi, Gunma, Chiba and Saga (Fig 1B). Races 2.5.9 and 4.5.9 that overcome *Cf-5* are confined to one prefecture only (Fig 1C) reflecting the locations where they were identified for the first time. So far, races 2.5.9 and 4.5.9 have not migrated to other prefectures. Although the *Cf-2* gene is no longer used in Japan, many race 2 isolates are still present throughout the country (Fig 1C), suggesting that these races are not outcompeted yet. From *Cf-0* plants not only race 0 isolates but often also additional races overcoming different *Cf* genes were isolated, including races 2, 4, 4.11 and 4.9.11 (Fig 2).

**Table 1:** The virulence spectrum of 133 isolates of *Cladosporium fulvum* collected in Japan as determined on a differential set of tomato cultivars carrying different *Cf* genes.

Resistance gene ^a^	Virulence spectrum of the isolates collected in Japan ^b^											
	0	2	4	9 ^c^	2.4	2.9 ^c^	2.5.9	4.9 ^c^	4.5.9 ^c^	4.11	2.4.11	4.9.11 ^c^
-	V	V	V	V	V	V	V	V	V	V	V	V
***Cf-2***	A	V	A	A	V	V	V	A	A	A	V	A
***Cf-4***	A	A	V	A	V	A	A	V	V	V	V	V
***Cf-5***	A	A	A	A	A	A	V	A	V	A	A	A
***Cf-6***	A	A	A	A	A	A	A	A	A	A	A	A
***Cf-9***	A	A	A	V	A	V	V	V	V	A	A	V
***Cf-4*, *Cf-11***	A	A	A	A	A	A	A	A	A	V	V	V
**Number of collected isolates**	12	15	17	1	2	8	2	5	4	46	5	16

^a^ A differential set of tomato cultivars including ‘Potentate’ (no *Cf* resistance gene), ‘Vetomold’ (*Cf-2*), ‘Purdue 135’ (*Cf-4*), ‘Moneymaker-Cf-5’ (*Cf-5*), ‘Ontario 7818’ (*Cf-6*), ‘Moneymaker-Cf-9’ (*Cf-9*), and ‘Ontario 7716’ (*Cf-4* and *Cf-11*) was used for inoculation to determine the virulence spectrum.

^b^ V, virulent on cultivar; A, avirulent on cultivar

^c^ Races unique to the Japanese *Cladosporium fulvum* population

Although *Cf-2*, *Cf-4*, *Cf-5* and *Cf-9* genes have all been employed in Japan, no isolates able to overcome all four *Cf* genes were identified (Table 1). Also no isolate overcoming the *Cf-6* gene was identified, which is consistent with the absence of this resistance gene in cultivars used in Japan.

### DNA modifications in *Avr* genes

The nucleotide sequences of the *Avr* genes of four race 0 isolates that served as a reference were considered to contain wild-type *Avr* genes [21]. The ability to overcome a particular *Cf* gene is likely attributed to non-synonymous DNA modification(s) in the ORF of a corresponding *Avr* gene or loss of the corresponding *Avr* gene. The *Avr2* gene amplified from races 2, 2.4, 2.9, 2.5.9 and 2.4.11 showed mutations that allowed them to overcome the *Cf-2-*mediated resistance (S2 Table). These races contain in total five different mutations in the *Avr2* ORF when compared with the wild-type reference *Avr2* gene including (i) a mutation destroying the start codon (c.1A>G; 21 isolates), (ii) a nucleotide insertion and substitution (c.50insT; c.52A>C; 4 isolates) leading to a truncated Avr2 protein, (iii) a nucleotide change (c.242G>T; 2 isolates) leading to Cys63Phe amino acid substitution in the C-terminus of the Avr2 protein, (iv) an insertion of five As (c. (64_69) insA; 1 isolate) leading to a frame shift in the Avr2 protein, and (v) a multiple nucleotide deletion (c.56delCAGCAGCCAA; 1 isolate) also leading to a frame shift in the Avr2 protein. Finally, a transposon insertion was observed in three isolates, leading to production of a nonfunctional Avr2 protein (S2 Table). Several of the observed mutations in Avr2 are new, and have not been observed in *C*. *fulvum* populations elsewhere in the world (Table 2).

**Table 2:** Overview of all DNA modifications present in the coding sequence of five avirulence genes in a population of 133 *Cladosporium fulvum* isolates collected in Japan.

*Avr* gene	Allele^a^	Frequency^b^	Effect on protein^c^	Remarks	Loss of *Cf*-mediated HR^d^	Genotypes with independent events ^e^
***Avr2***	Wild-type	101			No (Fig 3)	
	**c.1A>G**	**21**	**p.Met1Val**	**Disruption of start codon**	**Yes**	**2 (G30, G37)**
	**c.50insT**	**(4)**	**p.Ile18ThrfsX24**	**Frameshift**	**Yes**	**2 (G03, G35)**
	**c.52A>C**	**(4)**	**p.Ile18Leu**	**Mutation in signal sequence**	**No**	
	**c.56delCAGCAGCCAA**	**1**	**p.Ala19GlufsX38**	**Frameshift**	**Yes**	**1 (G31)**
	c. (64–69)insA	1	p.leu24TyrfsX18	Frameshift	Yes	1 (G11)
	**c.242G>T**	**2**	**p.Cys63Phe**	**Disruption of S-bridge**	**Yes (Fig 3)**	**1 (G08)**
	Transposon insertion	3	no protein		Yes	1 (G01)
***Avr4***	Wild-type	38			No (Fig 3)	
	**c.118T>C**	**1**	**p.Cys40Arg**	**Disruption of S-bridge**	**Yes (Fig 3)**	**1 (G04)**
	**c.191G>T**	**48**	**p.Cys64Phe**	**Disruption of S-bridge**	**Yes (Fig 3)**	**4 (G19, G20, G26, G32)**
	**c.191G>C**	**34**	**p.Cys64Ser**	**Disruption of S-bridge**	**Yes (Fig 3)**	**1 (G38)**
	c.191G>A	2	p.Cys64Tyr	Disruption of S-bridge	Yes (Fig 3)	1 (G08)
	**c.318delG**	**10**	**p.Ser107ValfsX4**	**Frameshift**	**Yes (Fig 3)**	**2 (G03, G13)**
***Avr4E***	Wild-type	31				
	c.244T>C;	(80)	p.Phe82Leu		Yes	
	c.278T>C	(80)	p.Met93Tyr		Yes	
	Gene deletion	22	no protein		Yes	1 (G27)
***Avr5***	Wild-type	127			No (Fig 3)	
	Gene deletion	4	no protein		Yes	2 (G16, G18)
	**c.268G>C**	**2**	**p.Gly90Arg**		**Yes (Fig 3)**	**1 (G15)**
***Avr9***	Wild-type	72				
	Gene deletion	36	no protein		Yes	5 (G06, G17, G24, G26, G40)
	C.23T>C	25	p.Val8Ala	Mutation in signal sequence	No	

^a ^Codes for mutations at DNA level are according to den Dunnen and Antonarakis [40]. Mutations specific to isolates of the Japanese population of *C*. *fulvum* are highlighted in bold.

^b^ The numbers refer to the number of isolates in the population carrying the allele. The DNA modifications in *Avr2* (c.50insT and c.52A>C) and *Avr4E* (c.244T>C and c.278T>C) that always appeared together were showed in parentheses.

^c^ Codes for mutations at protein level are according to den Dunnen and Antonarakis [40]. Mutations specific to isolates of the Japanese population of *C*. *fulvum* are highlighted in bold.

^d ^The *Avr* alleles were expressed in the PVX expression system and analyzed in tomato plants carrying the corresponding *Cf* resistance gene. Representative pictures of the hypersensitive response (HR)-inducing activity of wild-type and mutant alleles are shown in Fig 3.

^e^ Single independent mutation, transposon insertion or deletion events deduced from related genotypes.

The *Avr4* gene amplified from races 4, 2.4, 4.9, 4.5.9, 4.11, 2.4.11 and 4.9.11 contained in total five different mutations in the *Avr4* ORF that allowed them to overcome the *Cf-4*-mediated resistance (S2 Table). They include (i) c.118T>C (1 isolate), (ii) c.191G> T (48 times), (iii) c.191G>C (34 isolates), (iv) c.191G>A (2 isolates), mutations all causing the substitution of a cysteine residue by an arginine, serine, phenylalanine and tyrosine residue, respectively. The mutation c.318delG causes a frame shift in the Avr4 protein (p. ser107ValfsX4; 10 isolates). Several of the observed mutations in *Avr4* gene are new and have not been observed in *C*. *fulvum* populations elsewhere in the world (Table 2).

The set of *Cf* differentials used to identify the virulence spectrum of the collection did not include a cultivar with the *Cf-4E* gene alone. *Cf-4E* is a paralog of the *Cf-4* gene and is located close to *Cf-4* gene, while effector genes including *Avr4* and *Avr4E* are not linked in the *C*. *fulvum* genome [14–17, 41]. In 80 out of 133 isolates two mutations (c.244T>C; c.278T>C) were observed in the *Avr4E* ORF causing two amino acid substitutions in the Avr4E protein (p.Leu82Phe and p.Thr93Met) (S2 Table) enabling them to overcome *Cf4-E*-mediated resistance [12]. Remarkably, 22 isolates that can overcome *Cf-4E*-mediated resistance in the Japanese *C*. *fulvum* population lacked the *Avr4E* gene. Both mutations in the *Avr4E* gene have been observed in *C*. *fulvum* populations elsewhere in the world [21].

Only six out of 133 isolates in the Japanese *C*. *fulvum* collection overcome *Cf-5*-mediated resistance (races 2.5.9 and 4.5.9) (Table 1). In two isolates of race 4.5.9, the *Avr5* gene contained a new mutation (c.268G>C) that had not been observed in *C*. *fulvum* populations elsewhere in the world (S2 Table). This new mutation involves the substitution of a glycine residue by a arginine in the C-terminus of the Avr5 protein (Table 2). The remaining four isolates of races 2.5.9 and 4.5.9 that did overcome *Cf-5*-mediated resistance had lost the *Avr5* gene (S2 Table). Overcoming Cf-5-mediated resistance by deletion of the *Avr5* gene has been reported before in *C*. *fulvum* [13].

Similarly, the *Avr9* gene in 36 isolates that overcome *Cf-9*-mediated resistance (races 9, 2.9, 2.5.9, 4.9, 4.5.9 and 4.9.11) was absent from the genome (S2 Table). In addition, in 25 isolates not able to overcome the *Cf-9* gene, the mutation c.23T>C in the *Avr9* ORF was observed leading to a p.Val8Ala amino acid substitution in the signal peptide of the Avr9 protein which has, however, no effect on its HR-inducing activity on *Cf-9* plants [21]. Overcoming the *Cf-9* resistance due to loss of the *Avr9* gene in *C*. *fulvum* has been observed before in populations of this fungus elsewhere in the world [21].

Overall, this molecular analysis revealed diverse types of mutations in *Avr* genes that reflect adaptation of *C*. *fulvum* to specific *Cf* genes.

### HR-inducing activity of novel mutant Avr proteins produced by new Japanese races overcoming *Cf*- mediated resistance

**Fig 3:**
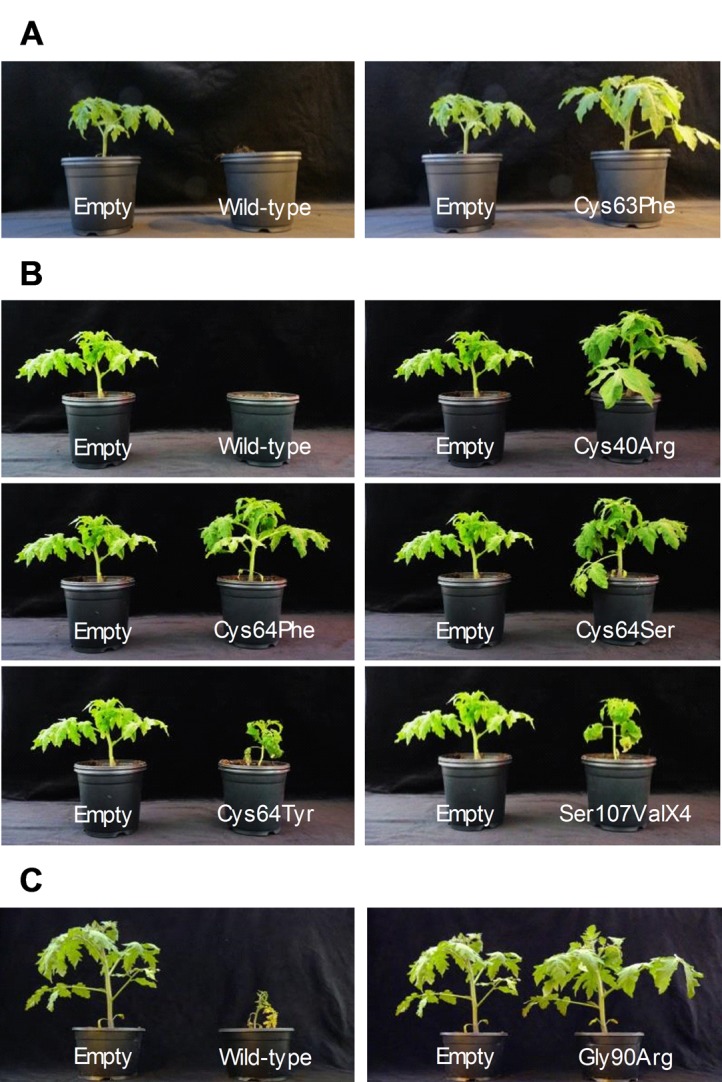
*Cf*-mediated hypersensitive responses (HR) triggered by wild-type and mutant Avr proteins. *Avr*-wild-type and mutant genes were cloned in the PVX vector and the recombinant PVX virus was assayed for HR-inducing activity on *Cf-2*, *Cf-4* or *Cf-5* tomato plants. (**A**) PVX-mediated expression of wild-type Avr2 protein causes strong HR-inducing activity and eventually kills *Cf-2* tomato plants, but mutant Avr2 protein (p.Cys63Phe substitution) present in isolate CF212 lost HR-inducing activity. (**B**) PVX-mediated expression of wild-type Avr4 protein causes strong HR-inducing activity and eventually kills *Cf-4* tomato plants. Five different mutant Avr4 proteins (substituted amino acid residues are indicated) all lost HR-inducing activity on *Cf-4* tomato plants. (**C**) PVX-mediated expression of wild-type Avr5 protein elicits a strong HR on *Cf-5* tomato plants, whereas mutant Avr5 protein (p.Gly90Arg substitution) lost HR-inducing activity. Plants were photographed at 3 weeks post inoculation.

All *Avr2*, *Avr4* and *Avr5* genes in the Japanese collection carrying novel DNA modifications in their ORFs were assayed for their HR-inducing activity on tomato plants carrying the corresponding *Cf* gene using the PVX expression system. *Avr* alleles carrying DNA modifications that were reported before [36] to have lost HR-inducing activity due to absence of an *Avr* gene (*Avr4E* and *Avr9*) or coding for a strongly truncated Avr protein (*Avr2*) were not included in these assays. *Avr* genes with novel DNA modifications were cloned in the PVX vector and assayed for HR-inducing activity on tomato plants carrying the corresponding *Cf* genes [36]. Without an exception, all *Avr* genes with novel and unique DNA modification in their *Avr* coding sequence had lost HR-inducing activity on tomato cultivars carrying the corresponding *Cf* gene (Fig 3).

Loss of *Cf-2*-mediated HR is usually caused by frame shifts observed in the *Avr2* gene [21], but here for the first time we observed an amino acid substitution in the Avr2 protein (p.Cys63Phe) that caused loss of HR-inducing activity on tomato plants carrying the *Cf-2* gene (Fig 3A).

Most of the novel mutations found in the *Avr4* gene led to cysteine substitutions in the Avr4 protein causing loss of HR-inducing activity on *Cf-4* plants (Fig 3B). The Cys64Tyr substitution in Avr4 reported before [42] showed a weaker effect on destroying HR-inducing activity on *Cf-4* plants than the novel Cys64Phe and Cys64Ser substitutions reported here. Also the new frame shift mutation (p.Ser107ValfsX4) caused loss of HR-inducing activity (Fig 3B).

Only the mutation (p. Pro4LeufsX18) in the signal peptide of the *Avr5* gene causing a frame shift in the Avr5 protein was reported previously [13].The amino acid substitution (p.Gly90Arg) in the Avr5 protein that caused loss of HR-inducing activity on tomato plants carrying the *Cf-5* gene had not been reported before (Fig 3C).

### Relationship between different *Avr* genotypes in *C*. *fulvum* isolates collected in Japan

In order to identify relationships between the *C*. *fulvum* isolates collected in Japan, we classified the different *Avr* genotypes based on the nucleotide sequence of *Avr* genes and presence of either the MAT1-1 or MAT1-2 mating type locus. In total 41 unique genotypes could be identified (G01 to G41; S2 Table). We subsequently tried to infer parental relationships between existing races and recently identified races 2.9, 4.9, 4.9.11 and 2.5.9 collected in same prefecture.

The race 2.9 isolates were first reported to occur in the Iwate prefecture [32] and most likely developed from a race 2 isolate present in Iwate. Race 2 isolate H-48/G07 from Iwate is most likely the parents of all race 2.9 isolates (G06) identified in Iwate (Table 3). Race 2 isolates occurring in other prefectures (Hokkaido, Akita, Fukushima, Gunma, Gifu, Mie, Shimane, Fukuoka, Nagasaki and Miyazaki) are excluded as the potential parents of race 2.9 isolates because they carry a different mating type locus or contain different mutations in ORFs, or low probability due to geographical distance (S2 Table; Fig 1). The other race 2.9 isolates were collected in prefectures Tochigi and Saga, but never a race 2 isolate has been collected in these prefectures (S2 Table).

**Table 3:** Potential parental isolates of new *Cladosporium fulvum* races collected in same prefecture.

Prefecture	Isolate	Genotype ^a^	MAT^b^	Race^c^	*Avr2* ^d^	*Avr4* ^d^	*Avr4E* ^d^	*Avr5* ^d^	*Avr9* ^d^
**(03) Iwate**	H-41	G06	MAT1-2	2.9	c.1A>G	c.-57G>A	c*36T>C	wild-type	**gene deletion**
	H-48^e^	G07	MAT1-2	2	c.1A>G	c.-57G>A	c*36T>C	wild-type	c.-103A>G; c.23T>C; c.*47A>C
**(09) Tochigi**	Ohtawara1	G14	MAT1-1	4.9	c.158+26_158+28insTGA	c.318delG	c.244T>C; c.278T>C; c.*36T>C	wild-type	**gene deletion**
	Ohtawara2	G14	MAT1-1	4.9	c.158+26_158+28insTGA	c.318delG	c.244T>C; c.278T>C; c.*36T>C	wild-type	**gene deletion**
	Kaminokawa^e^	G13	MAT1-1	4	c.158+26_158+28insTGA	c.318delG	c.244T>C; c.278T>C; c.*36T>C	wild-type	c.-103A>G; c.23T>C; c.*47A>C
**(10) Gunma**	CF308	G12	MAT1-1	4.9.11	c.158+26_158+28insTGA	c.191G>T	c.244T>C; c.278T>C; c.*36T>C	wild-type	**gene deletion**
	CF309	G12	MAT1-1	4.9.11	c.158+26_158+28insTGA	c.191G>T	c.244T>C; c.278T>C; c.*36T>C	wild-type	**gene deletion**
	CF307^e^	G20	MAT1-1	4.11	c.158+26_158+28insTGA	c.191G>T	c.244T>C; c.278T>C; c.*36T>C	wild-type	wild-type
**(09) Tochigi**	Utsunomiya1	G18	MAT1-1	2.5.9	c.1A>G; c.158+26_158+28insTGA	wild-type	**c.244T>C; c.278T>C;** c.*36T>C	**gene deletion**	gene deletion
	Utsunomiya2	G18	MAT1-1	2.5.9	c.1A>G; c.158+26_158+28insTGA	wild-type	**c.244T>C; c.278T>C;** c.*36T>C	**gene deletion**	gene deletion
	Tochigi1^e^	G17	MAT1-1	2.9	c.1A>G; c.158+26_158+28insTGA	wild-type	c*36T>C	wild-type	gene deletion
	Tochigi2^e^	G17	MAT1-1	2.9	c.1A>G; c.158+26_158+28insTGA	wild-type	c*36T>C	wild-type	gene deletion

^a^ Genotype of isolates based on sequence of *Avr* genes and mating type loci.

^b^ Mating type of isolate; MAT1-1 or MAT1-2.

^c^ Virulence spectrum of isolate.

^d^ DNA modifications observed in *Avr* genes; Codes for mutations at DNA level are according to den Dunnen and Antonarakis (2000) [40]. Mutations different to potential parental isolate are highlighted in bold.

^e^ Potential parental isolate(s)

Race 4.9 isolates were first reported to occur in Gunma prefecture and race 4.9.11 isolates in prefectures Gunma, Chiba and Fukushima [31] and this study discovered race 4.9 isolates in Tochigi (S2 Table). No additional race 4.9 and 4.9.11 isolates were identified outside these prefectures. Thus, race 4.9 and 4.9.11 isolates have likely developed from parental race 4 and race 4.11 isolates, respectively. By comparing the genotypes of the new race 4.9 and race 4.9.11 isolates with potential parental race 4 and race 4.11 isolates, they all appear to carry MAT1-1 and all contain the c.158+26_158+28insTGA mutation in the *Avr2* gene, c.*36T>C mutation in the *Avr4E* gene and wild-type *Avr5* gene. Race 4.9 stains (G14) from Tochigi were most likely derived from a race 4 isolate Kaminokawa/G13 identified from Tochigi (Table 3). For the new race 4.9.11 isolates collected in Gunma, the parent is most likely race 4.11 isolate CF307/G20 collected in same prefecture Gunma (Table 3).

Isolates that can overcome *Cf-5*-mediated resistance were recently collected and races 2.5.9 and 4.5.9 were identified only in Tochigi prefecture [33]. Thus, the new race 2.5.9 and 4.5.9 isolates have likely developed from regional parental race 2.9 and 4.9 isolates, respectively. Race 2.9 isolates (G17) from Tochigi are most likely the parents of race 2.5.9 stains (G18) identified in Tochigi based on mating type locus and nucleotide sequences of *Avr* genes (Table 3). Potential parental isolate of races 9 and 4.5.9 isolated from same prefecture could not be assigned.

## Discussion

### Strong selection pressure on *C*. *fulvum* population by tomato *Cf* resistance genes

*C*. *fulvum* is present in Japan since the 1920s, but introduction of most *Cf* resistance genes in tomato plants started later than elsewhere in the world [21, 34]. The *Cf-2* gene has been introduced in tomato grown in Japan in the 1960s [27], the *Cf-4* and *Cf-9* genes in the last two decades, and the *Cf-5* gene recently. Since the introduction of the latter *Cf* genes, no nation-wide survey has been performed in Japan; little is known about changes in the virulence spectrum of the *C*. *fulvum* population in Japan, whereas nothing is known about DNA modifications in *Avr* genes of isolates overcoming introduced *Cf* genes.

A total of 133 isolates collected between 1997 and 2013 belong to 12 different races (Table 1). Only four races (0, 2, 2.4, and 2.4.11) were reported to occur in Japan before 1998 [29]. Introductions of the *Cf-4* gene in the late 1990s and the *Cf-9* gene in the 2000s and very recently the *Cf-5* gene has led to the appearance of eight new races that can overcome the *Cf-2*, *Cf-4*, *Cf-5* or *Cf-9* gene (races 4, 2.9, 2.5.9, 4.9, 4.5.9, 4.11 and 4.9.11 [30–33]; race 9 identified in this report). Of these twelve races, seven have a virulence spectrum also reported to occur elsewhere in the world [21, 34], whereas five have a virulence spectrum that is unique to Japan (races 9, 2.9, 4.9, 4.5.9 and 4.9.11). Most of the DNA modifications observed in the *Avr* genes of the races that can overcome *Cf-2-*, *Cf-4-*, *Cf-5-* or *Cf-9-*mediated resistance are unique to the Japanese population of *C*. *fulvum* (Table 2). Strikingly, mutations in *Avr2* and *Avr4* genes are very diverse (6 and 5 different types, respectively), suggesting that new races may have arisen independently from different parental isolates. Similarly, *Avr9* deletion in races overcoming *Cf-9* gene occurred at least five times independently, which is at much higher frequency than deletion of *Avr4E* and *Avr5*, or transposon insertion in *Avr2* that occurred only once in the studied population (Table 2). The frequency of *Avr* gene loss is likely correlated with the presence of repeats flanking these *Avr* genes causing their instability [42].

Introduction of single *Cf* genes not only contributed to the appearance of new races, but also decreased the genetic diversity of the fungal population. The fact that race 4.11 isolates rather than race 4 isolates seem to have been selected recently by introduction of *Cf-4* cultivars in central Japan suggests that growers also used cultivars that contained the *Cf-11* gene (Fig 1). Surprisingly, although the race 2 isolates are still collected frequently, very few race 2.4 and 2.4.11 isolates were collected, suggesting that simultaneous adaptation to both the *Cf-2* and *Cf-4* might cause a fitness penalty.

### New races of *C*. *fulvum* adapted to *Cf-2*, *Cf-4*, *Cf-5* and *Cf-9* genes carry unique mutations in the corresponding *Avr* genes

The majority (71%) of the collected isolates in the population can overcome the *Cf-4* gene, 50% the *Cf-11* gene, 27% the *Cf-9* gene, 24% the *Cf-2* gene and 4.5% the *Cf-5* gene. From *Cf-0* tomato cultivars, not only race 0 isolates but often also isolates adapted to different *Cf* genes were collected (Fig 2). This is surprising as *Avr* genes are supposed to encode virulence factors, and races with a complex virulence spectrum are supposed to be less viable on *Cf-0* plants than race 0 isolates and would be outcompeted by the latter in time [23, 43–45]. DNA modification in *Avr* genes leading to virulence would be beneficial for a isolate only when growing on cultivars carrying the corresponding *Cf* gene, except for modifications that avoid recognition by the corresponding Cf proteins without affecting their virulence function. The latter is true for isolates that overcome the *Cf-4* gene as they produce mutated versions of Avr4 proteins that are no longer recognized by the Cf-4 protein but can still bind to chitin and protect the fungus against the deleterious effects of plant chitinases [46–49]. Most of DNA modifications identified in the *Avr4* gene of Japanese isolates adapted to Cf-4 plants are unique, lead to production of an unstable Avr4 effector that still binds to chitin. This mechanism of avoiding Cf-4 recognition seems to be under strong selection because the same position (c. 191G) in the *Avr4* gene showed three different nucleotide substitutions that occurred at least six times (Table 2). Another unique DNA modification observed in the *Avr4* gene (c.318delG) causes a frame shift at the 3’ of the gene leading to a mutant Avr4 protein that is 25 amino acid residues shorter than the wild-type protein and is no longer recognized by the Cf-4 protein (Table 2; Fig 3). Frame shift mutations in Avr4 leading to a truncated Avr4 protein have reported only once before [41], but it is not known whether these truncated versions of Avr4 are still able to bind chitin. Dissociation between chitin binding and recognition by the Cf-4 protein might explain why such a high frequency of isolates that can overcome the *Cf-4* gene occurs. These isolates are likely not quickly outcompeted by race 0 isolates on *Cf-0* tomato plants.

In race 2 isolates that can overcome *Cf-2*-mediated resistance, the mechanism might be different as they no longer produce a functional Avr2 protein that can inhibit plant cysteine proteases (the supposedly intrinsic virulence function of Avr2) like Rcr3, Pip2, TD65 and aleurain present in the apoplast of tomato [50, 51]. The c.A>G mutation leading to disruption of the start codon, and the c.242G>T mutation destroying a disulfide bond at the C-terminus of the Avr2 protein are most conspicuous. Interestingly, the latter mutation has been introduced artificially before in Avr2, and the shorter but stable Avr2 protein lacking the C-terminal disulfide bond produced by this mutant was hundred fold less active than the wild-type Avr2 protein in inhibiting Rcr3 cysteine protease and triggering Cf-2-mediated HR [50]. It is surprising that none of the cultivars from which isolates were collected contained the *Cf-2* gene, whereas still 24% of the collected isolates can still overcome the *Cf-2* resistance gene (Table 1). As the intrinsic function of the *Avr2* gene is lost in race 2 isolates, it is expected that on *Cf-0* plants race 2 isolates will gradually be outcompeted by race 0 isolates as they are supposed to be less viable.

Interestingly, all Japanese isolates that can overcome the *Cf-9* resistance gene lacked the entire *Avr9* gene which was also reported for this type of isolates collected elsewhere in the world [9, 21]. The genome of *C*. *fulvum* has recently been sequenced and the *Avr9* gene was shown to be flanked by repeats, which might explain *Avr9* instability leading to its loss [42]. Loss of the *Avr9* gene seems to occur even at a higher frequency than the accumulation of DNA modification in the *Avr9* gene as all *Avr9* genes present in isolates that could not overcome the *Cf-9* gene appeared identical. Although the biological function of the Avr9 protein is not known, it is expected that races lacking the *Avr9* gene would also be outcompeted in time by race 0 isolates on *Cf-0* plants since the *Avr9* gene is supposed to encode virulence factor as well. Similarly, deletion of the *Avr4E* gene was also observed and might be explained by its location in a repeat-rich area in the genome [42].

Likewise all isolates that overcome *Cf-5* resistance gene collected elsewhere in the world had lost the entire *Avr5* gene, except isolate IPO 1979 which has a frame shift mutation in the signal peptide leading to a pseudogene [13]. This study revealed that all isolates that could not overcome *Cf-5* resistance gene possessed a wild-type *Avr5* gene without any DNA modification, and two isolates of race 4.5.9 with a glycine to arginine substitution in C-terminus of Avr5 protein caused loss of HR-inducing activity on tomato plants carrying the *Cf-5* gene (Fig 3). These results imply that accumulation of mutations in the *Avr5* gene occur less frequently than loss of the gene from the genome of *C*. *fulvum*. Like the *Avr9*, the *Avr5* gene in the genome of *C*. *fulvum* is also surrounded by repetitive elements, which might explain *Avr5* gene instability leading to its loss [13]. The *Avr5* gene functions as a virulence factor, but the intrinsic function of the Avr5 protein is not known yet [13].

Although the Japanese population of *C*. *fulvum* has evolved independently from populations elsewhere in the world, it is striking that all unique DNA modifications observed in the *Avr* genes leading to adaptation to different *Cf* genes have similar biological effects on the encoded effector proteins [9, 12, 21].

Following similar overall mechanisms of adaptation in *C*. *fulvum* populations independently in different parts of the world might be related to the intrinsic functions of the Avr proteins, or their indirect or direct interaction with corresponding Cf proteins. Avr2 interacts indirectly with the Cf-2 protein through inhibition of Rcr3, which triggers a Cf-2-mediated HR [50–52]. Avr9 is also expected to interact indirectly with the Cf-9 protein [53]. A high affinity binding site is assumed to be the target of Avr9 which is guarded by the Cf-9 protein. Indirect interaction of an Avr protein with the corresponding R protein favors jettison of the Avr-encoding gene as has been observed for *Avr4E* and *Avr9* [12, 54, 55]. Point mutations in an Avr protein in order to overcome recognition by a matching Cf resistance protein might suggest direct interaction with that Cf protein as is suggested for the Avr4 protein for which the Cf-4 resistance protein is supposed to be the only host plant target [36].

### Prospects for protection of Japanese tomato cultivars against new races of *C*. *fulvum*

Based on the 41 genotypes identified in the Japanese population of *C*. *fulvum*, for only the most recently identified races the potential parental isolate could be inferred. The oldest races 0 and 2 isolates carry more diverse genotypes (16) and are more widespread in Japan than the new races that overcome the recently introduced *Cf* genes. The relatively new race 4, 4.11, 2.4 and 2.4.11 isolates overcoming the *Cf-4* gene that was introduced in the 1990s have spread over a large part of the country. Especially race 4.11 isolates have migrated into many different prefectures, suggesting that adaptation to both the *Cf-4* and *Cf-11* genes does not strongly affect viability of these isolates.

The very new race 9, 2.9, 2.5.9, 4.9, 4.5.9 and 4.9.11 isolates were mainly confined to one prefecture or a small region only. Overall, new races do not seem to spread very quickly, likely due to the fact that tomatoes are grown in greenhouses limiting the possibilities of conidia to escape.

Presently, most commercial tomato cultivars in Japan contain only single *Cf* genes, mainly the *Cf-4*, *Cf-5* or *Cf-9* resistance gene, but the high frequency of races overcoming these *Cf* genes has become a serious problem for tomato growers since 1990s [29–33]. The quick appearance of new race isolates is likely due to sequential introduction of single *Cf* genes in e monocultures of tomato cultivars. In addition, as the use of agrochemicals in Japan has been reduced significantly since 1990s, control of *C*. *fulvum* largely depends on resistance cultivars carrying *Cf* genes only. This has increased the selection pressure on this pathogen which has led to serious disease outbreaks caused by races with new virulence spectra [29–33]. Combining multiple *Cf* genes in one cultivar, as has occurred in most European countries, has slowed down the appearance of new races with complex virulence spectra. So far the new complex races mainly occur locally, introduction of multiple *Cf* genes (*Cf-2*, *Cf-4*, *Cf-5* and *Cf-9*) in one cultivar would slow down the spread of these races. These *Cf* genes could also be combined with the *Cf-6* gene as so far in Japan no race 6 isolates have been identified even elsewhere in the world. Also different *Cf-Ecp* genes could be introduced as worldwide no isolates of *C*. *fulvum* have been reported with *Avr6* or *Ecp* genes adapted to these *Cf* genes [45, 56, 57].

## Supporting Information

S1 Table Primers used in this study (DOCX)

S2 Table Information on 133 isolates of *Cladosporium fulvum* collected in Japan. (DOCX)

## References

[1] Thomma BPHJ, Van Esse HP, De Wit PJGM. *Cladosporium fulvum* (syn. *Passalora fulva*), a highly specialized plant pathogen as a model for functional studies on plant pathogenic Mycosphaerellaceae. Mol Plant Pathol. 2005;6: 379–393. 10.1111/j.1364-3703.2005.00292.x 20565665

[2] Cooke MC. New American fungi. Grivillea XII: 32; 1883.

[3] De Wit PJGM. A light and scanning-electron microscopic study of the infection of tomato plants by virulent and avirulent races of *Cladosporium fulvum* . Neth J Plant Pathol. 1977;83: 109–122.

[4] Lazarovits G, Higgins VJ. Histological comparison of *Cladosporium fulvum* race 1 on immune, resistant and susceptible tomato varieties. Can J Bot. 1976;54: 224–234.

[5] Lazarovits G, Higgins VJ. Ultrastucture of susceptible, resistant and immune reactions of tomato to races of *Cladosporium fulvum* . Can J Bot. 1976;54: 235–249.

[6] Stergiopoulos I, De Wit PJGM. Fungal effector proteins. Ann Rev Phytopathol. 2009;47: 233–263. 10.1146/annurev.phyto.112408.132637 19400631

[7] Joosten MHAJ, Cozijnsen TJ, De Wit PJGM. Host resistance to a fungal tomato pathogen lost by a single base-pair change in an avirulence gene. Nature. 1994;367: 384–386. 8114941 10.1038/367384a0

[8] Luderer R, De Kock MJD, Dees RHL, De Wit PJGM, Joosten MHAJ. Functional analysis of cysteine residues of Ecp elicitor proteins of the fungal tomato pathogen *Cladosporium fulvum* . Mol Plant Pathol. 2002;3: 91–95. 10.1046/j.1464-6722.2001.00095.x 20569313

[9] Van den Ackerveken GFJM, Van Kan JAL, De Wit PJGM. Molecular analysis of the avirulence gene *Avr9* of the fungal tomato pathogen *Cladosporium fulvum* fully supports the gene-for-gene hypothesis. Plant J. 1992;2: 359–366. 1303800 10.1111/j.1365-313x.1992.00359.x

[10] Van Kan JAL, Van den Ackerveken GFJM, De Wit PJGM. Cloning and characterization of cDNA of avirulence gene *Avr9* of the fungal pathogen *Cladosporium fulvum*, causal agent of tomato leaf mold. Mol Plant Microbe Interact. 1991;4: 52–59. 1799694 10.1094/mpmi-4-052

[11] Westerink N, Joosten MHAJ, De Wit PJGM. Fungal (a)virulence factors at the crossroads of disease susceptibility and resistance In: Punja ZK, editor. Fungal disease resistance in plants: Biochemistry, Molecular Biology, and Genetic Engineering. Binghamton: Food Products Press; 2004 pp. 93–137.

[12] Westerink N, Brandwagt BF, De Wit PJGM, Joosten MHAJ. *Cladosporium fulvum* circumvents the second functional resistance gene homologue at the *Cf-4* locus (Hcr9-4E) by secretion of a stable avr4E isoform. Mol Microbiol. 2004;54: 533–545. 15469522 10.1111/j.1365-2958.2004.04288.x

[13] Mesarich CH, Griffiths SA, Van der Burgt A, Ӧkmen B, Beenen HG, Etalo DW, et al Transcriptome sequencing uncovers the *Avr5* avirulence gene of the tomato leaf mold pathogen *Cladosporium fulvum* . Mol Plant Microbe Interact. 2014;27: 846–857. 10.1094/MPMI-02-14-0050-R 24678832

[14] Dixon MS, Jones DA, Keddie JS, Thomas CM, Harrison K, Jones JDG. The tomato *Cf-2* disease resistance locus comprises two functional genes encoding leucine-rich repeat proteins. Cell. 1996;84: 451–459. 8608599 10.1016/s0092-8674(00)81290-8

[15] Jones DA, Thomas CM, Hammond-Kosack KE, Balint-Kurti PJ, Jones JDG. Isolation of the tomato *Cf-9* gene for resistance to *Cladosporium fulvum* by transposon tagging. Science. 1994;266: 789–793. 7973631 10.1126/science.7973631

[16] Takken FLW, Thomas CM, Joosten MHAJ, Golstein C, Westerink N, Hille J, et al A second gene at the tomato *Cf-4* locus confers resistance to *Cladosporium fulvum* through recognition of a novel avirulence determinant. Plant J. 1999;20: 279–288. 10571888 10.1046/j.1365-313x.1999.t01-1-00601.x

[17] Thomas CM, Jones DA, Parniske M, Harrison K, Balint-Kurti PJ, Hatzixanthis K, et al Characterization of the tomato *Cf-4* gene for resistance to *Cladosporium fulvum* identifies sequences that determine recognitional specificity in *Cf-4* and *Cf-9* . Plant Cell. 1997;9: 2209–2224. 9437864 10.1105/tpc.9.12.2209 PMC157069

[18] Dixon MS, Hatzixanthis K, Jones DA, Harrison K, Jones JDG. The tomato *Cf-5* disease resistance gene and six homologs show pronounced allelic variation in leucine-rich repeat copy number. Plant Cell. 1998;10: 1915–1925. 9811798 10.1105/tpc.10.11.1915 PMC143956

[19] Rivas S, Thomas CM. Molecular interactions between tomato and the leaf mold pathogen *Cladosporium fulvum* . Ann Rev Phytopathol. 2005;43: 395–436. 16078890 10.1146/annurev.phyto.43.040204.140224

[20] Joosten MHAJ, De Wit PJGM. The tomato-*Cladosporium fulvum* interaction: A versatile experimental system to study plant-pathogen interactions. Ann Rev Phytopathol. 1999;37: 335–367. 11701827 10.1146/annurev.phyto.37.1.335

[21] Stergiopoulos I, De Kock MJD, Lindhout P, De Wit PJGM. Allelic variation in the effector genes of the tomato pathogen *Cladosporium fulvum* reveals different modes of adaptive evolution. Mol Plant Microbe Interact. 2007;20: 1271–1283. 17918629 10.1094/MPMI-20-10-1271

[22] De Wit PJGM, Joosten MHAJ, Thomma BPHJ, Stergiopoulos I. Gene-for-gene models and beyond: the *Cladosporium fulvum*-tomato pathosystem In: Deising HB, editor. Plant Relationships. The Mycota V. Berlin Heidelberg: Springer; 2009 pp. 135–156.

[23] De Wit PJGM, Mehrabi R, Van den Burg HA, Stergiopoulos I. Fungal effector proteins: past, present and future. Mol Plant Pathol. 2009;10: 735–747. 10.1111/j.1364-3703.2009.00591.x 19849781 PMC6640362

[24] Kishi K. Studies on the physiological specialization of *Cladosporium fulvum* Cooke. Japanease J Phytopathol. 1962;27: 189–196.

[25] Kooistra E. Recent experiences breeding leaf mould resistant tomatoes. Euphytica. 1964;13: 103–109.

[26] Langford AN. The parasitism of *Cladosporium fulvum* Cooke and the genetics of resistance to it. Can J Research, Section C. 1937;15: 109–128.

[27] Sugahara Y, Suzuki I, Tokada S, Kuriyama T. Sudies on breeding tomatoes for leaf mold resistance. II. Characters of new tomato selections highly resistanr to leaf mold and breeding procedure. Bulletin Horticultural Research Station Series B. 1965;4: 17–36.

[28] Fujimori M, Kobayashi T, Baba H. Pathogenicity of leaf mould and resistance of tomato cultivars in Nagano prefecture. J Japan Soc Hort Sci. 1980;49: 208–209.

[29] Yamada K, Abiko K. Race composition of *Fulvia fulva* in Japan during 1997–1998. Japanese J Phytopathol. 2002;68: 36–38.

[30] Satou M, Shinozaki T, Nishi K, Kubota M. Leaf mold of tomato caused by races 4 and 4.11 of *Passalora fulva* in Japan. J Gen Plant Pathol. 2005;71: 436–437.

[31] Enya J, Ikeda K, Takeuchi T, Horikoshi N, Higashi T, Sakai T, et al The first occurrence of leaf mold of tomato caused by races 4.9 and 4.9.11 of *Passalora fulva* (syn. *Fulvia fulva*) in Japan. J Gen Plant Pathol. 2009;75: 76–79.

[32] Iida Y, Iwadate Y, Kubota M, Terami F. Occurrence of a new race 2.9 of leaf mold of tomato in Japan. J Gen Plant Pathol. 2010;76: 84–86.

[33] Kubota M, Morishima M, Iida Y. First occurrence of tomato leaf mold caused by race 2.5.9 and 4.5.9 of the pathogen, *Passalora fulva*, in Japan. J Gen Plant Pathol. 2015; In press.

[34] Lindhout P, Korta W, Cislik M, Vos I, Gerlagh T. Further Identification of races of *Cladosporium fulvum* (*Fulvia fulva*) on tomato originating from the Netherlands, France and Poland. Neth J Plant Pathol. 1989;95: 143–148.

[35] Kishi K, Abiko K. Studies on the physiological specialization of *Cladosporium fulvum* Cooke. II. Racial identification of isolates collected from 11 prefectures in Japan from 1971 to 1973. Japanease J Phytopathol. 1976;42: 497–499.

[36] Stergiopoulos I, Van den Burg HA, Ökmen B, Beenen H, Kema GHJ, De Wit PJGM. Tomato Cf resistance proteins mediate recognition of cognate homologous effectors from fungi pathogenic on dicots and monocots. Proc Natl Acad Sci USA. 2010;107: 7610–7615. 10.1073/pnas.1002910107 20368413 PMC2867746

[37] Van der Hoorn RAL, Laurent F, Roth R, De Wit PJGM. Agroinfiltration is a versatile tool that facilitates comparative analyses of Avr9/Cf-9-induced and Avr4/Cf-4-induced necrosis. Mol Plant Microbe Interact. 2000;13: 439–446. 10755307 10.1094/MPMI.2000.13.4.439

[38] Holiday P, Mulder JL. *Fulvia fulva*. In: CMI descriptions of pathogenic fungi and bacteria. No. 487. Commonwealth Mycological Institute, Kew, Surrey, UK; 1976.

[39] Stergiopoulos I, Groenewald M, Staats M, Lindhout P, Crous PW, De Wit PJGM. Mating-type genes and the genetic structure of a world-wide collection of the tomato pathogen *Cladosporium fulvum* . Fungal Genet Biol. 2007;44: 415–429. 17178244 10.1016/j.fgb.2006.11.004

[40] Den Dunnen JT, Antonarakis SE. Mutation nomenclature extensions and suggestions to describe complex mutations: A discussion. Hum Mutat. 2000;15: 7–12. 10612815 10.1002/(SICI)1098-1004(200001)15:1<7::AID-HUMU4>3.0.CO;2-N

[41] De Wit PJGM, Van der Burgt A, Ökmen B, Stergiopoulos I, Abd-Elsalam KA, Aerts AL, et al. The genomes of the fungal plant pathogens *Cladosporium fulvum* and *Dothistroma septosporum* reveal adaptation to different hosts and lifestyles but also signatures of common ancestry. PLOS Genetics. 2012; 10.137:1003088. 10.1371/journal.pgen.1003088 PMC3510045 23209441

[42] Joosten MH, Vogelsang R, Cozijnsen TJ, Verberne MC, De Wit PJ. The biotrophic fungus *Cladosporium fulvum* circumvents *Cf-4*-mediated resistance by producing unstable *AVR4* elicitors. Plant Cell. 1997;9: 367–379. 9090881 10.1105/tpc.9.3.367 PMC156924

[43] Bolton MD, Van Esse HP, Vossen JH, De Jonge R, Stergiopoulos I, Stulemeijer IJ, et al The novel *Cladosporium fulvum* lysin motif effector Ecp6 is a virulence factor with orthologues in other fungal species. Mol Microbiol. 2008;69: 119–136. 10.1111/j.1365-2958.2008.06270.x 18452583

[44] De Jonge R, Van Esse HP, Kombrink A, Shinya T, Desaki Y, Bours R, et al Conserved fungal LysM effector Ecp6 prevents chitin-triggered immunity in plants. Science. 2010;329: 953–955. 10.1126/science.1190859 20724636

[45] Laugé R, De Wit PJGM. Fungal avirulence genes: structure and possible functions. Fungal Genet Biol. 1998;24: 285–297. 9756710 10.1006/fgbi.1998.1076

[46] Van den Burg HA, Harrison SJ, Joosten MHAJ, Vervoort J, De Wit PJGM. *Cladosporium fulvum* Avr4 protects fungal cell walls against hydrolysis by plant chitinases accumulating during infection. Mol Plant Microbe Interact. 2006;19: 1420–1430. 17153926 10.1094/MPMI-19-1420

[47] Van den Burg HA, Spronk CAEM, Boeren S, Kennedy MA, Vissers JPC, Vuister GW, et al Binding of the Avr4 elicitor of *Cladosporium fulvum* to chitotriose units is facilitated by positive allosteric protein-protein interactions: the chitin-binding site of Avr4 represents a novel binding site on the folding scaffold shared between the invertebrate and the plant chitin-binding domain. J Biol Chem. 2004;279: 16786–16796. 14769793 10.1074/jbc.M312594200

[48] Van den Burg HA, Westerink N, Francoijs KJ, Roth R, Woestenenk E, Boeren S, et al Natural disulfide bond-disrupted mutants of Avr4 of the tomato pathogen *Cladosporium fulvum* are sensitive to proteolysis, circumvent Cf-4-mediated resistance, but retain their chitin-binding ability. J Biol Chem. 2003;278: 27340–27346. 12736265 10.1074/jbc.M212196200

[49] Van Esse HP, Bolton MD, Stergiopoulos I, De Wit PJGM, Thomma BPHJ. The chitin-binding *Cladosporium fulvum* effector protein Avr4 is a virulence factor. Mol Plant Microbe Interact. 2007;20: 1092–1101. 17849712 10.1094/MPMI-20-9-1092

[50] Van 't Klooster JW, Van der Kamp MW, Vervoort J, Beekwilder J, Boeren S, Joosten MHAJ, et al Affinity of Avr2 for tomato cysteine protease Rcr3 correlates with the Avr2-triggered *Cf-2*-mediated hypersensitive response. Mol Plant Pathol. 2011;12: 21–30. 10.1111/j.1364-3703.2010.00647.x 21118346 PMC6640376

[51] Van Esse HP, Van ‘t Klooster JW, Bolton MD, Yadeta KA, Van Baarlen P, Boeren S, et al The *Cladosporium fulvum* virulence protein Avr2 inhibits host proteases required for basal defense. Plant Cell. 2008;20: 1948–1963. 10.1105/tpc.108.059394 18660430 PMC2518240

[52] Rooney HCE, Van ‘t Klooster JW, Van der Hoorn RAL, Joosten MHAJ, Jones JDG, De Wit PJGM. Cladosporium Avr2 inhibits tomato Rcr3 protease required for *Cf-2*-dependent disease resistance. Science. 2005;308: 1783–1786. 15845874 10.1126/science.1111404

[53] Kooman-Gersmann M, Honée G, Bonnema G, De Wit PJGM. A high-affinity binding site for the Avr9 peptide elicitor of *Cladosporium fulvum* is present on plasma membranes of tomato and other solanaceous plants. Plant Cell. 1996;8: 929–938. 12239406 10.1105/tpc.8.5.929 PMC161149

[54] Jones JDG, Dangl JL. The plant immune system. Nature. 2006;444: 323–329. 17108957 10.1038/nature05286

[55] Kooman-Gersmann M, Vogelsang R, Vossen P, Van den Hooven HW, Mahé E, Honée G, et al Correlation between binding affinity and necrosis-inducing activity of mutant Avr9 peptide elicitors. Plant Physiol. 1998;117: 609–618. 9625714 10.1104/pp.117.2.609 PMC34981

[56] Laugé R, Goodwin PH, De Wit PJGM, Joosten MHAJ. Specific HR-associated recognition of secreted proteins from *Cladosporium fulvum* occurs in both host and non-host plants. Plant J. 2000;23: 735–745. 10998185 10.1046/j.1365-313x.2000.00843.x

[57] Laugé R, Joosten MHAJ, Haanstra JPW, Goodwin PH, Lindhout P, De Wit PJGM. Successful search for a resistance gene in tomato targeted against a virulence factor of a fungal pathogen. Proc Natl Acad Sci USA. 1998;95: 9014–9018. 9671796 10.1073/pnas.95.15.9014 PMC21194

