# Supplementary material for: Novel Mutations Detected in Avirulence Genes Overcoming Tomato Cf Resistance Genes in Isolates of a Japanese Population of Cladosporium fulvum
Source: PLoS One. 2015 Apr 22;10(4):e0123271. doi: 10.1371/journal.pone.0123271 (PMC4406682; doi:10.1371/journal.pone.0123271)
Supplement: S1 Table — (DOCX) [file pone.0123271.s001.docx]

**Table S1. Primers used in this study**

| **Primer code** | **Gene-specific primers (5′-3′)** |
| --- | --- |
| RM-Avr2-F | GCGGACAAATCATCAGCATA |
| RM-Avr2-R | AGGCAGATCTGGGTACAAGC |
| RM-Avr4-F | ACGCAGGTCCAAAATAGCTC |
| RM-Avr4-R | TCGCAGTTATTTCACCTTGCT |
| RM-Avr4E-F | CCGCAGCGAAGTAAATTTTG |
| RM-Avr4E-R | GTCAGTCCAGTCCGGAACC |
| RM-Avr5_F1 | CGTTACAGCAGTCCGAAAGC |
| RM-Avr5_R1 | TCGTCCCTACCCGGTTCTTA |
| RM-Avr9-F | AGTAGATCCGGCCGAGAGAG |
| RM-Avr9-R | AAAGCCTTCAATATGAACGAAT |
| M13Avr2-F | GTAAAACGACGGCCAGTGCGGACAATCATCAGCATATTCTC |
| M13Avr2-R | GCGGATAACAATTTCACACAGGGAGGCAGATCTGGGTACAAGCGAAG |
| M13Avr4-F | GTAAAACGACGGCCAGTCATACGCAGGTCCAAAATAGCTCAGG |
| M13Avr4-R | GCGGATAACAATTTCACACAGGTCGCAGTTATTTCACCTTGCTGG |
| M13Avr4E-F | GTAAAACGACGGCCAGTCCGCAGCGAAGTAAATTTTG |
| M13Avr4E-R | GCGGATAACAATTTCACACAGGCTTGCTGTCTGTCAGTCCAGTCC |
| M13FAvr9-F | GTAAAACGACGGCCAGTGAGAGAGAGATATACAGGTATAAG |
| M13RAvr9-R | GCGGATAACAATTTCACACAGGGACCAAAAGCCTTCAATATGAACGAATG |
| MAT1-1 P1F | CTTCACCACACCCAAAC |
| MAT1-1 P4R | TGTTCGGTGTCGTGATG |
| MAT1-2 P4R | TCCACGTCGAAGTAGAG |
| MAT1-2 P1F | CTGCCAGTTCTGCTTTG |
| Attb1_AscI_PR1A_F | GGGGACAAGTTTGTACAAAAAAGCAGGCTGGCGCGCCATGGGATTTGTTCTCTTTTCA |
| PR1A-R | ATTTTGGGCACGGCAAGAG |
| PR1A_Avr2_F | CTTGCCGTGCCCAAAATGCAGCCAAAAAACTACCTGGC |
| Avr2-Int-F2 | GCTCGGCGACATGTGACGGGAAGTTGACGAGG |
| Avr2-Int-R2 | CCTCGTCAACTTCCCGTCACATGTCGCCGAGC |
| Attb2_XhoI_Avr2_R | GGGGACCACTTTGTACAAGAAAGCTGGGTCTCGAGTCAACCGCAAAGACCAAA |
| PR1A_Avr4_F | CTTGCCGTGCCCAAAATAAGGCCCCCAAAACTCAA |
| Attb2_XhoI_Avr4_R | GGGGACCACTTTGTACAAGAAAGCTGGGTCTCGAGTCATTGCGGCGTCTT |
| Avr4_318delG_R | GGGGACCACTTTGTACAAGAAAGCTGGGTCTCGAGCTTTACCGGA |
| Avr5-Haga-F1-1 | TGCTCAAAAATACGCTCGCCCACG |
| Avr5-Haga-R1-1 | GTATTTTTGAGCAACATCTGGCATCGC |
