# Supplementary material for: Novel Mutations Detected in Avirulence Genes Overcoming Tomato Cf Resistance Genes in Isolates of a Japanese Population of Cladosporium fulvum
Source: PLoS One. 2015 Apr 22;10(4):e0123271. doi: 10.1371/journal.pone.0123271 (PMC4406682; doi:10.1371/journal.pone.0123271)
Supplement: S2 Table — (DOCX) [file pone.0123271.s002.docx]

**Table S2. Information on 133 isolates of *Cladosporium fulvum* collected in Japan.**

| Strain code^a^ | Geno-type^b^ | Date^c^ | Prefecture ^d^ | *Cf* ^e^ | MAT^f^ | Race ^g^ | DNA modification of *Avr* genes ^h^ | | | | |
| --- | --- | --- | --- | --- | --- | --- | --- | --- | --- | --- | --- |
|  |  |  |  |  |  |  | *Avr2* | *Avr4* | *Avr4E* | *Avr5* | *Avr9* |
| CF110 | G01 | 1997 | (01) Hokkaido | - | MAT1-1 | 2 | transposon insertion | c.-57G>A | c*36T>C | wild-type | wild-type |
| ktk1 | G02 | 2005 | (01) Hokkaido | *Cf-0* | MAT1-1 | 0 | c.158+26_158+28insTGA | c.-57G>A | c*36T>C | wild-type | c.-103A>G; c.23T>C; c.*47A>C |
| ktm1 | G03 | 2005 | (01) Hokkaido | *Cf-4* | MAT1-2 | 2.4 | c.50insT; c.52A>C; c.158+26_158+28insTGA | c.318delG | c.244T>C; c.278T>C; c.*36T>C | wild-type | c.-103A>G; c.23T>C; c.*47A>C |
| sng1 | G04 | 2005 | (01) Hokkaido | *Cf-4* | MAT1-1 | 2.4.11 | transposon insertion | c.-57G>A; c.118T>C | c.244T>C; c.278T>C; c.*36T>C | wild-type | c.-103A>G; c.23T>C; c.*47A>C |
| sy2 | G05 | 2005 | (01) Hokkaido | *Cf-0* | MAT1-1 | 0 | c.158+26_158+28insTGA | wild-type | c.244T>C; c.278T>C; c.*36T>C | wild-type | c.-103A>G; c.23T>C; c.*47A>C |
| H-28 | G06 | 2008 | (03) Iwate | *Cf-9* | MAT1-2 | 2.9 | c.1A>G | c.-57G>A | c*36T>C | wild-type | gene deletion |
| H-29 | G06 | 2008 | (03) Iwate | *Cf-9* | MAT1-2 | 2.9 | c.1A>G | c.-57G>A | c*36T>C | wild-type | gene deletion |
| H-41 | G06 | 2008 | (03) Iwate | *Cf-9* | MAT1-2 | 2.9 | c.1A>G | c.-57G>A | c*36T>C | wild-type | gene deletion |
| H-7 | G06 | 2008 | (03) Iwate | *Cf-9* | MAT1-2 | 2.9 | c.1A>G | c.-57G>A | c*36T>C | wild-type | gene deletion |
| H-9 | G06 | 2008 | (03) Iwate | *Cf-9* | MAT1-2 | 2.9 | c.1A>G | c.-57G>A | c*36T>C | wild-type | gene deletion |
| H-48 | G07 | 2009 | (03) Iwate | *-* | MAT1-2 | 2 | c.1A>G | c.-57G>A | c*36T>C | wild-type | c.-103A>G; c.23T>C; c.*47A>C |
| CF212 | G08 | 1997 | (04) Miyagi | - | MAT1-2 | 2.4.11 | c.242G>T; c.158+26_158+28insTGA | c.191G>A | c.244T>C; c.278T>C; c.*36T>C | wild-type | c.-103A>G; c.23T>C; c.*47A>C |
| AP22 | G09 | 2007 | (05) Akita | *Cf-0* | MAT1-1 | 2 | transposon insertion | c.-57G>A | c.244T>C; c.278T>C; c.*36T>C | wild-type | c.-103A>G; c.23T>C; c.*47A>C |
| CF108 | G07 | 1997 | (07) Fukushima | - | MAT1-2 | 2 | c.1A>G | c.-57G>A | c*36T>C | wild-type | c.-103A>G; c.23T>C; c.*47A>C |
| Fukushima4 | G07 | 2004 | (07) Fukushima | - | MAT1-2 | 2 | c.1A>G | c.-57G>A | c*36T>C | wild-type | c.-103A>G; c.23T>C; c.*47A>C |
| Fukushima7 | G10 | 2004 | (07) Fukushima | - | MAT1-1 | 2 | c.1A>G | c.-57G>A | c.244T>C; c.278T>C; c.*36T>C | wild-type | c.-103A>G; c.23T>C; c.*47A>C |
| Fukushima8 | G11 | 2004 | (07) Fukushima | - | MAT1-2 | 2 | c. (64_69)insA; c.158+26_158+28insTGA | c.-57G>A | c*36T>C | wild-type | c.-103A>G; c.23T>C; c.*47A>C |
| Nango1 | G12 | 2007 | (07) Fukushima | *Cf-9* | MAT1-1 | 4.9.11 | c.158+26_158+28insTGA | c.191G>T | c.244T>C; c.278T>C; c.*36T>C | wild-type | gene deletion |
| Nango4 | G12 | 2007 | (07) Fukushima | *Cf-9* | MAT1-1 | 4.9.11 | c.158+26_158+28insTGA | c.191G>T | c.244T>C; c.278T>C; c.*36T>C | wild-type | gene deletion |
| Yabuki | G12 | 2007 | (07) Fukushima | *Cf-9* | MAT1-1 | 4.9.11 | c.158+26_158+28insTGA | c.191G>T | c.244T>C; c.278T>C; c.*36T>C | wild-type | gene deletion |
| Kaminokawa | G13 | 2013 | (09) Tochigi | - | MAT1-1 | 4 | c.158+26_158+28insTGA | c.318delG | c.244T>C; c.278T>C; c.*36T>C | wild-type | c.-103A>G; c.23T>C; c.*47A>C |
| Ohtawara1 | G14 | 2013 | (09) Tochigi | *Cf-9* | MAT1-1 | 4.9 | c.158+26_158+28insTGA | c.318delG | c.244T>C; c.278T>C; c.*36T>C | wild-type | gene deletion |
| Ohtawara2 | G14 | 2013 | (09) Tochigi | *Cf-9* | MAT1-1 | 4.9 | c.158+26_158+28insTGA | c.318delG | c.244T>C; c.278T>C; c.*36T>C | wild-type | gene deletion |
| Haga1 | G15 | 2013 | (09) Tochigi | *Cf-5, Cf-9* | MAT1-1 | 4.5.9 | c.158+26_158+28insTGA | c.191G>T | c.244T>C; c.278T>C; c.*36T>C | c.268G>C | gene deletion |
| Haga2 | G15 | 2013 | (09) Tochigi | *Cf-5, Cf-9* | MAT1-1 | 4.5.9 | c.158+26_158+28insTGA | c.191G>T | c.244T>C; c.278T>C; c.*36T>C | c.268G>C | gene deletion |
| Moka1 | G16 | 2013 | (09) Tochigi | *Cf-5, Cf-9* | MAT1-1 | 4.5.9 | c.158+26_158+28insTGA | c.191G>T | c.244T>C; c.278T>C; c.*36T>C | gene deletion | gene deletion |
| Moka2 | G16 | 2013 | (09) Tochigi | *Cf-5, Cf-9* | MAT1-1 | 4.5.9 | c.158+26_158+28insTGA | c.191G>T | c.244T>C; c.278T>C; c.*36T>C | gene deletion | gene deletion |
| Tochigi1 | G17 | 2013 | (09) Tochigi | - | MAT1-1 | 2.9 | c.1A>G; c.158+26_158+28insTGA | wild-type | c*36T>C | wild-type | gene deletion |
| Tochigi2 | G17 | 2013 | (09) Tochigi | - | MAT1-1 | 2.9 | c.1A>G; c.158+26_158+28insTGA | wild-type | c*36T>C | wild-type | gene deletion |
| Utsunomiya1 | G18 | 2013 | (09) Tochigi | *Cf-5, Cf-9* | MAT1-1 | 2.5.9 | c.1A>G; c.158+26_158+28insTGA | wild-type | c.244T>C; c.278T>C; c.*36T>C | gene deletion | gene deletion |
| Utsunomiya2 | G18 | 2013 | (09) Tochigi | *Cf-5, Cf-9* | MAT1-1 | 2.5.9 | c.1A>G; c.158+26_158+28insTGA | wild-type | c.244T>C; c.278T>C; c.*36T>C | gene deletion | gene deletion |
| CF305 | G12 | 2008 | (10) Gunma | *Cf-9* | MAT1-1 | 4.9.11 | c.158+26_158+28insTGA | c.191G>T | c.244T>C; c.278T>C; c.*36T>C | wild-type | gene deletion |
| CF308 | G12 | 2008 | (10) Gunma | *Cf-9* | MAT1-1 | 4.9.11 | c.158+26_158+28insTGA | c.191G>T | c.244T>C; c.278T>C; c.*36T>C | wild-type | gene deletion |
| CF309 | G12 | 2008 | (10) Gunma | *Cf-9* | MAT1-1 | 4.9.11 | c.158+26_158+28insTGA | c.191G>T | c.244T>C; c.278T>C; c.*36T>C | wild-type | gene deletion |
| CF318 | G12 | 2008 | (10) Gunma | *Cf-9* | MAT1-1 | 4.9.11 | c.158+26_158+28insTGA | c.191G>T | c.244T>C; c.278T>C; c.*36T>C | wild-type | gene deletion |
| CF325 | G12 | 2008 | (10) Gunma | *Cf-9* | MAT1-1 | 4.9.11 | c.158+26_158+28insTGA | c.191G>T | c.244T>C; c.278T>C; c.*36T>C | wild-type | gene deletion |
| CF326 | G12 | 2008 | (10) Gunma | *Cf-9* | MAT1-1 | 4.9.11 | c.158+26_158+28insTGA | c.191G>T | c.244T>C; c.278T>C; c.*36T>C | wild-type | gene deletion |
| 334 | G19 | 2007 | (10) Gunma | *Cf-9* | MAT1-1 | 4.9 | c.158+26_158+28insTGA | c.191G>T | c.244T>C; c.278T>C; c.*36T>C | wild-type | gene deletion |
| 338 | G19 | 2007 | (10) Gunma | *Cf-9* | MAT1-1 | 4.9 | c.158+26_158+28insTGA | c.191G>T | c.244T>C; c.278T>C; c.*36T>C | wild-type | gene deletion |
| 342 | G19 | 2007 | (10) Gunma | *Cf-9* | MAT1-1 | 4.9 | c.158+26_158+28insTGA | c.191G>T | c.244T>C; c.278T>C; c.*36T>C | wild-type | gene deletion |
| CF307 | G20 | 2008 | (10) Gunma | *Cf-0* | MAT1-1 | 4.11 | c.158+26_158+28insTGA | c.191G>T | c.244T>C; c.278T>C; c.*36T>C | wild-type | wild-type |
| CF310 | G21 | 2008 | (10) Gunma | *Cf-0* | MAT1-1 | 2 | c.1A>G | c.-57G>A | c*36T>C | wild-type | c.-103A>G; c.23T>C; c.*47A>C |
| CF315 | G22 | 2008 | (10) Gunma | *Cf-0* | MAT1-2 | 4.11 | c.158+26_158+28insTGA | c.191G>T | c.244T>C; c.278T>C; c.*36T>C | wild-type | wild-type |
| CF319 | G23 | 2008 | (10) Gunma | *Cf-0* | MAT1-2 | 0 | c.158+26_158+28insTGA | wild-type | c.244T>C; c.278T>C; c.*36T>C | wild-type | wild-type |
| CF323 | G24 | 2008 | (10) Gunma | *Cf-9* | MAT1-1 | 9 | c.158+26_158+28insTGA | wild-type | c.244T>C; c.278T>C; c.*36T>C | wild-type | gene deletion |
| CF282 | G22 | 2008 | (11) Saitama | *Cf-0* | MAT1-2 | 4.11 | c.158+26_158+28insTGA | c.191G>T | c.244T>C; c.278T>C; c.*36T>C | wild-type | wild-type |
| CF285 | G22 | 2008 | (11) Saitama | *Cf-0* | MAT1-2 | 4.11 | c.158+26_158+28insTGA | c.191G>T | c.244T>C; c.278T>C; c.*36T>C | wild-type | wild-type |
| CF289 | G25 | 2008 | (11) Saitama | *Cf-4* | MAT1-2 | 4.11 | c.158+26_158+28insTGA | c.191G>C | c*36T>C | wild-type | wild-type |
| C1 | G12 | 2008 | (12) Chiba | *Cf-0* | MAT1-1 | 4.9.11 | c.158+26_158+28insTGA | c.191G>T | c.244T>C; c.278T>C; c.*36T>C | wild-type | gene deletion |
| Chi1 | G12 | 2007 | (12) Chiba | *Cf-9* | MAT1-1 | 4.9.11 | c.158+26_158+28insTGA | c.191G>T | c.244T>C; c.278T>C; c.*36T>C | wild-type | gene deletion |
| Chi3 | G12 | 2007 | (12) Chiba | *Cf-9* | MAT1-1 | 4.9.11 | c.158+26_158+28insTGA | c.191G>T | c.244T>C; c.278T>C; c.*36T>C | wild-type | gene deletion |
| P1 | G12 | 2008 | (12) Chiba | *Cf-9* | MAT1-1 | 4.9.11 | c.158+26_158+28insTGA | c.191G>T | c.244T>C; c.278T>C; c.*36T>C | wild-type | gene deletion |
| P3 | G12 | 2008 | (12) Chiba | *Cf-9* | MAT1-1 | 4.9.11 | c.158+26_158+28insTGA | c.191G>T | c.244T>C; c.278T>C; c.*36T>C | wild-type | gene deletion |
| HM1 | G25 | 2008 | (12) Chiba | Cf-0 | MAT1-2 | 4.11 | c.158+26_158+28insTGA | c.191G>C | c*36T>C | wild-type | wild-type |
| A1 | G26 | 2008 | (12) Chiba | *Cf-9* | MAT1-1 | 4.9.11 | c.158+26_158+28insTGA | c.191G>T | c*36T>C | wild-type | gene deletion |
| A2 | G26 | 2008 | (12) Chiba | *Cf-9* | MAT1-1 | 4.9.11 | c.158+26_158+28insTGA | c.191G>T | c*36T>C | wild-type | gene deletion |
| HM2 | G27 | 2008 | (12) Chiba | *Cf-0* | MAT1-2 | 4.11 | c.158+26_158+28insTGA | c.191G>C | gene deletion | wild-type | wild-type |
| CF230 | G08 | 1998 | (14) Kanagawa | - | MAT1-2 | 2.4.11 | c.242G>T; c.158+26_158+28insTGA | c.191G>A | c.244T>C; c.278T>C; c.*36T>C | wild-type | c.-103A>G; c.23T>C; c.*47A>C |
| CF334 | G28 | 2008 | (16) Toyama | *Cf-4* | MAT1-1 | 4.11 | c.158+26_158+28insTGA | c.318delG | c.244T>C; c.278T>C; c.*36T>C | wild-type | c.-103A>G; c.23T>C; c.*47A>C |
| CF335 | G29 | 2008 | (16) Toyama | *Cf-4* | MAT1-2 | 4.11 | c.158+26_158+28insTGA | c.191G>C | c.244T>C; c.278T>C; c.*36T>C | wild-type | wild-type |
| GF922 | G22 | 2007 | (21) Gifu | *Cf-0* | MAT1-2 | 4.11 | c.158+26_158+28insTGA | c.191G>T | c.244T>C; c.278T>C; c.*36T>C | wild-type | wild-type |
| GF926 | G22 | 2007 | (21) Gifu | *Cf-0* | MAT1-2 | 4.11 | c.158+26_158+28insTGA | c.191G>T | c.244T>C; c.278T>C; c.*36T>C | wild-type | wild-type |
| GF927 | G22 | 2007 | (21) Gifu | *Cf-0* | MAT1-2 | 4.11 | c.158+26_158+28insTGA | c.191G>T | c.244T>C; c.278T>C; c.*36T>C | wild-type | wild-type |
| GF932 | G22 | 2007 | (21) Gifu | *Cf-0* | MAT1-2 | 4.11 | c.158+26_158+28insTGA | c.191G>T | c.244T>C; c.278T>C; c.*36T>C | wild-type | wild-type |
| GF941 | G22 | 2007 | (21) Gifu | *Cf-0* | MAT1-2 | 4.11 | c.158+26_158+28insTGA | c.191G>T | c.244T>C; c.278T>C; c.*36T>C | wild-type | wild-type |
| GF929 | G25 | 2007 | (21) Gifu | *Cf-0* | MAT1-2 | 4.11 | c.158+26_158+28insTGA | c.191G>C | c*36T>C | wild-type | wild-type |
| GF940 | G30 | 2007 | (21) Gifu | *Cf-0* | MAT1-1 | 2 | c.1A>G | c.-57G>A | c*36T>C | wild-type | wild-type |
| CF296 | G22 | 2008 | (22) Shizuoka | *Cf-4* | MAT1-2 | 4.11 | c.158+26_158+28insTGA | c.191G>T | c.244T>C; c.278T>C; c.*36T>C | wild-type | wild-type |
| CF294 | G31 | 2008 | (22) Shizuoka | *Cf-0* | MAT1-1 | 0 | c.158+26_158+28insTGA | wild-type | c.244T>C; c.278T>C; c.*36T>C | wild-type | wild-type |
| CF278 | G20 | 2008 | (23) Aichi | *Cf-0* | MAT1-1 | 4.11 | c.158+26_158+28insTGA | c.191G>T | c.244T>C; c.278T>C; c.*36T>C | wild-type | wild-type |
| CF280 | G27 | 2008 | (23) Aichi | *Cf-0* | MAT1-2 | 4.11 | c.158+26_158+28insTGA | c.191G>C | gene deletion | wild-type | wild-type |
| CF267 | G32 | 2008 | (23) Aichi | *Cf-0* | MAT1-2 | 4 | c.158+26_158+28insTGA | c.191G>T | c.244T>C; c.278T>C; c.*36T>C | wild-type | wild-type |
| CF271 | G32 | 2008 | (23) Aichi | *Cf-4* | MAT1-2 | 4 | c.158+26_158+28insTGA | c.191G>T | c.244T>C; c.278T>C; c.*36T>C | wild-type | wild-type |
| CF269 | G33 | 2008 | (23) Aichi | *Cf-0* | MAT1-2 | 0 | c.158+26_158+28insTGA | wild-type | c*36T>C | wild-type | c.-103A>G; c.23T>C; c.*47A>C |
| CF270 | G34 | 2008 | (23) Aichi | *Cf-0* | MAT1-2 | 4 | c.158+26_158+28insTGA | c.191G>C | gene deletion | wild-type | wild-type |
| CF275 | G34 | 2008 | (23) Aichi | *Cf-4* | MAT1-2 | 4 | c.158+26_158+28insTGA | c.191G>C | gene deletion | wild-type | wild-type |
| CF277 | G34 | 2008 | (23) Aichi | *Cf-4* | MAT1-2 | 4 | c.158+26_158+28insTGA | c.191G>C | gene deletion | wild-type | wild-type |
| CF371 | G20 | 2008 | (24) Mie | Cf-0 | MAT1-1 | 4.11 | c.158+26_158+28insTGA | c.191G>T | c.244T>C; c.278T>C; c.*36T>C | wild-type | wild-type |
| CF344 | G22 | 2008 | (24) Mie | *Cf-0* | MAT1-2 | 4.11 | c.158+26_158+28insTGA | c.191G>T | c.244T>C; c.278T>C; c.*36T>C | wild-type | wild-type |
| CF348 | G22 | 2008 | (24) Mie | *Cf-0* | MAT1-2 | 4.11 | c.158+26_158+28insTGA | c.191G>T | c.244T>C; c.278T>C; c.*36T>C | wild-type | wild-type |
| CF353 | G22 | 2008 | (24) Mie | *Cf-0* | MAT1-2 | 4.11 | c.158+26_158+28insTGA | c.191G>T | c.244T>C; c.278T>C; c.*36T>C | wild-type | wild-type |
| CF357 | G22 | 2008 | (24) Mie | *Cf-0* | MAT1-2 | 4.11 | c.158+26_158+28insTGA | c.191G>T | c.244T>C; c.278T>C; c.*36T>C | wild-type | wild-type |
| CF359 | G22 | 2008 | (24) Mie | *Cf-0* | MAT1-2 | 4.11 | c.158+26_158+28insTGA | c.191G>T | c.244T>C; c.278T>C; c.*36T>C | wild-type | wild-type |
| CF373 | G22 | 2008 | (24) Mie | *Cf-0* | MAT1-2 | 4.11 | c.158+26_158+28insTGA | c.191G>T | c.244T>C; c.278T>C; c.*36T>C | wild-type | wild-type |
| CF375 | G22 | 2008 | (24) Mie | *Cf-4* | MAT1-2 | 4.11 | c.158+26_158+28insTGA | c.191G>T | c.244T>C; c.278T>C; c.*36T>C | wild-type | wild-type |
| CF380 | G23 | 2008 | (24) Mie | Cf-0 | MAT1-2 | 0 | c.158+26_158+28insTGA | wild-type | c.244T>C; c.278T>C; c.*36T>C | wild-type | wild-type |
| CF342 | G27 | 2008 | (24) Mie | *Cf-0* | MAT1-2 | 4.11 | c.158+26_158+28insTGA | c.191G>C | gene deletion | wild-type | wild-type |
| CF363 | G27 | 2008 | (24) Mie | *Cf-0* | MAT1-2 | 4.11 | c.158+26_158+28insTGA | c.191G>C | gene deletion | wild-type | wild-type |
| CF365 | G27 | 2008 | (24) Mie | *Cf-0* | MAT1-2 | 4.11 | c.158+26_158+28insTGA | c.191G>C | gene deletion | wild-type | wild-type |
| CF351 | G28 | 2008 | (24) Mie | *Cf-0* | MAT1-1 | 4.11 | c.158+26_158+28insTGA | c.318delG | c.244T>C; c.278T>C; c.*36T>C | wild-type | c.-103A>G; c.23T>C; c.*47A>C |
| CF377 | G28 | 2008 | (24) Mie | *Cf-0* | MAT1-1 | 4.11 | c.158+26_158+28insTGA | c.318delG | c.244T>C; c.278T>C; c.*36T>C | wild-type | c.-103A>G; c.23T>C; c.*47A>C |
| CF367 | G34 | 2008 | (24) Mie | *Cf-0* | MAT1-2 | 4 | c.158+26_158+28insTGA | c.191G>C | gene deletion | wild-type | wild-type |
| CF381 | G34 | 2008 | (24) Mie | *Cf-0* | MAT1-2 | 4 | c.158+26_158+28insTGA | c.191G>C | gene deletion | wild-type | wild-type |
| CF169 | G35 | 1997 | (24) Mie | *-* | MAT1-1 | 2.4.11 | c.50insT; c.52A>C; c.158+26_158+28insTGA | c.318delG | c.244T>C; c.278T>C; c.*36T>C | wild-type | c.-103A>G; c.23T>C; c.*47A>C |
| CF361 | G36 | 2008 | (24) Mie | *Cf-0* | MAT1-2 | 2 | c.56delCAGCAGCCAAA; c.158+26_158+28insTGA | c.-57G>A | c.244T>C; c.278T>C; c.*36T>C | wild-type | wild-type |
| CF369 | G37 | 2008 | (24) Mie | *Cf-0* | MAT1-2 | 2 | c.1A>G | c.-57G>A | c*36T>C | wild-type | wild-type |
| CF257 | G05 | 2008 | (27) Osaka | *Cf-0* | MAT1-1 | 0 | c.158+26_158+28insTGA | wild-type | c.244T>C; c.278T>C; c.*36T>C | wild-type | c.-103A>G; c.23T>C; c.*47A>C |
| CF256 | G27 | 2008 | (27) Osaka | *Cf-0* | MAT1-2 | 4.11 | c.158+26_158+28insTGA | c.191G>C | gene deletion | wild-type | wild-type |
| CF258 | G27 | 2008 | (27) Osaka | *Cf-4* | MAT1-2 | 4.11 | c.158+26_158+28insTGA | c.191G>C | gene deletion | wild-type | wild-type |
| CF254 | G32 | 2008 | (27) Osaka | *Cf-0* | MAT1-2 | 4 | c.158+26_158+28insTGA | c.191G>T | c.244T>C; c.278T>C; c.*36T>C | wild-type | wild-type |
| CF255 | G38 | 2008 | (27) Osaka | *Cf-0* | MAT1-2 | 4 | c.158+26_158+28insTGA | c.191G>C | c*36T>C | wild-type | wild-type |
| CF265 | G22 | 2008 | (28) Hyogo | *Cf-0* | MAT1-2 | 4.11 | c.158+26_158+28insTGA | c.191G>T | c.244T>C; c.278T>C; c.*36T>C | wild-type | wild-type |
| CF333 | G25 | 2008 | (28) Hyogo | *Cf-4* | MAT1-2 | 4.11 | c.158+26_158+28insTGA | c.191G>C | c*36T>C | wild-type | wild-type |
| CF263 | G34 | 2008 | (28) Hyogo | *Cf-4* | MAT1-2 | 4 | c.158+26_158+28insTGA | c.191G>C | gene deletion | wild-type | wild-type |
| CF264 | G34 | 2008 | (28) Hyogo | *Cf-4* | MAT1-2 | 4 | c.158+26_158+28insTGA | c.191G>C | gene deletion | wild-type | wild-type |
| CF330 | G34 | 2008 | (28) Hyogo | *Cf-4* | MAT1-2 | 4 | c.158+26_158+28insTGA | c.191G>C | gene deletion | wild-type | wild-type |
| CF250 | G32 | 2008 | (30) Wakayama | *Cf-0* | MAT1-2 | 4 | c.158+26_158+28insTGA | c.191G>T | c.244T>C; c.278T>C; c.*36T>C | wild-type | wild-type |
| CF247 | G39 | 2008 | (30) Wakayama | *Cf-0* | MAT1-1 | 0 | c.158+26_158+28insTGA | c.-57G>A | c.244T>C; c.278T>C; c.*36T>C | wild-type | c.-103A>G; c.23T>C; c.*47A>C |
| CF249 | G39 | 2008 | (30) Wakayama | *Cf-0* | MAT1-1 | 0 | c.158+26_158+28insTGA | c.-57G>A | c.244T>C; c.278T>C; c.*36T>C | wild-type | c.-103A>G; c.23T>C; c.*47A>C |
| KO-2 | G25 | 2008 | (31) Tottori | *Cf-4* | MAT1-2 | 4.11 | c.158+26_158+28insTGA | c.191G>C | c*36T>C | wild-type | wild-type |
| KO-3 | G27 | 2008 | (31) Tottori | *Cf-4* | MAT1-2 | 4.11 | c.158+26_158+28insTGA | c.191G>C | gene deletion | wild-type | wild-type |
| KO-4 | G27 | 2008 | (31) Tottori | *Cf-4* | MAT1-2 | 4.11 | c.158+26_158+28insTGA | c.191G>C | gene deletion | wild-type | wild-type |
| KO-5 | G27 | 2008 | (31) Tottori | *Cf-4* | MAT1-2 | 4.11 | c.158+26_158+28insTGA | c.191G>C | gene deletion | wild-type | wild-type |
| KO-1 | G38 | 2008 | (31) Tottori | *Cf-4* | MAT1-2 | 4 | c.158+26_158+28insTGA | c.191G>C | c*36T>C | wild-type | wild-type |
| CF159 | G30 | 1997 | (32) Shimane | - | MAT1-1 | 2 | c.1A>G | c.-57G>A | c*36T>C | wild-type | wild-type |
| CF303 | G38 | 2008 | (33) Okayama | *Cf-0* | MAT1-2 | 4 | c.158+26_158+28insTGA | c.191G>C | c*36T>C | wild-type | wild-type |
| CF328 | G23 | 2008 | (36) Tokushima | *Cf-0* | MAT1-2 | 0 | c.158+26_158+28insTGA | wild-type | c.244T>C; c.278T>C; c.*36T>C | wild-type | wild-type |
| 11-1 | G03 | 2003 | (38) Ehime | *-* | MAT1-2 | 2.4 | c.50insT; c.52A>C; c.158+26_158+28insTGA | c.318delG | c.244T>C; c.278T>C; c.*36T>C | wild-type | c.-103A>G; c.23T>C; c.*47A>C |
| 3-2 | G35 | 2003 | (38) Ehime | *-* | MAT1-1 | 2.4.11 | c.50insT; c.52A>C; c.158+26_158+28insTGA | c.318delG | c.244T>C; c.278T>C; c.*36T>C | wild-type | c.-103A>G; c.23T>C; c.*47A>C |
| CF300 | G23 | 2008 | (40) Fukuoka | *Cf-0* | MAT1-2 | 0 | c.158+26_158+28insTGA | wild-type | c.244T>C; c.278T>C; c.*36T>C | wild-type | wild-type |
| CF298 | G29 | 2008 | (40) Fukuoka | *Cf-0* | MAT1-2 | 4.11 | c.158+26_158+28insTGA | c.191G>C | c.244T>C; c.278T>C; c.*36T>C | wild-type | wild-type |
| CF299 | G30 | 2008 | (40) Fukuoka | *Cf-0* | MAT1-1 | 2 | c.1A>G | c.-57G>A | c*36T>C | wild-type | wild-type |
| CF259 | G27 | 2008 | (41) Saga | *Cf-0* | MAT1-2 | 4.11 | c.158+26_158+28insTGA | c.191G>C | gene deletion | wild-type | wild-type |
| CF262 | G27 | 2008 | (41) Saga | *Cf-0* | MAT1-2 | 4.11 | c.158+26_158+28insTGA | c.191G>C | gene deletion | wild-type | wild-type |
| CF261 | G40 | 2008 | (41) Saga | *Cf-9* | MAT1-1 | 2.9 | c.1A>G | c.-57G>A | c.244T>C; c.278T>C; c.*36T>C | wild-type | gene deletion |
| CF251 | G30 | 2008 | (42) Nagasaki | *Cf-0* | MAT1-1 | 2 | c.1A>G | c.-57G>A | c*36T>C | wild-type | wild-type |
| Aso2 | G38 | 2007 | (43) Kumamoto | *-* | MAT1-2 | 4 | c.158+26_158+28insTGA | c.191G>C | c*36T>C | wild-type | wild-type |
| T1 | G05 | 2008 | (45) Miyazaki | *Cf-0* | MAT1-1 | 0 | c.158+26_158+28insTGA | wild-type | c.244T>C; c.278T>C; c.*36T>C | wild-type | c.-103A>G; c.23T>C; c.*47A>C |
| M4-1 | G22 | 2008 | (45) Miyazaki | *Cf-0* | MAT1-2 | 4.11 | c.158+26_158+28insTGA | c.191G>T | c.244T>C; c.278T>C; c.*36T>C | wild-type | wild-type |
| MC3 | G27 | 2008 | (45) Miyazaki | *Cf-0* | MAT1-2 | 4.11 | c.158+26_158+28insTGA | c.191G>C | gene deletion | wild-type | wild-type |
| MC9 | G27 | 2008 | (45) Miyazaki | *Cf-0* | MAT1-2 | 4.11 | c.158+26_158+28insTGA | c.191G>C | gene deletion | wild-type | wild-type |
| M3-6 | G29 | 2008 | (45) Miyazaki | *Cf-0* | MAT1-2 | 4.11 | c.158+26_158+28insTGA | c.191G>C | c.244T>C; c.278T>C; c.*36T>C | wild-type | wild-type |
| MC8 | G41 | 2008 | (45) Miyazaki | *Cf-0* | MAT1-2 | 2 | c.1A>G | c.-57G>A | c.244T>C; c.278T>C; c.*36T>C | wild-type | wild-type |

^a^ Acronym given to the isolate when collected.

^b^ Genotype of isolates based on sequence of *Avr* genes and mating type loci.

^c^ Year of collection of isolate.

^d^ Prefecture from where isolate was collected.

^e^ *Cf* resistance gene present in cultivar from which isolate was collected.

^f^ Mating type of isolate; MAT1-1 or MAT1-2.

^g^ Virulence spectrum of isolate.

^h^ DNA modifications observed in *Avr* genes; Codes for mutations at DNA level are according to den Dunnen and Antonarakis (2000).
